# Synthesis of Ni-Doped Tremolite Fibers to Help Clarify the Aetiology of the Cytotoxic Outcome of Asbestos

**DOI:** 10.3390/nano13081303

**Published:** 2023-04-07

**Authors:** Andrea Bloise, Eugenia Giorno, Domenico Miriello, Nicolas Godbert

**Affiliations:** 1 Department of Biology, Ecology and Earth Sciences, University of Calabria, 87036 Rende (CS), Italy; 2 University Museum System—SiMU, Section of Mineralogy and Petrography, University of Calabria, 87036 Arcavacata di Rende (CS), Italy; 3 MAT-INLab Laboratory of Inorganic Molecular Materials, Department of Chemistry and Chemical Technologies, University of Calabria, 87036 Rende (CS), Italy

**Keywords:** asbestos, tremolite, nickel, hydrothermal synthesis, tremolite Raman spectroscopy

## Abstract

Asbestos fibers act as complex crystal-chemical reservoirs susceptible of releasing potentially toxic elements (such as ions impurities) into the lung cellular environment during permanency and dissolution. To comprehend the exact pathological mechanisms that are triggered upon inhalation of asbestos fibers, in vitro studies on possible interactions between the mineral and the biological system have been carried out mostly by using natural asbestos. However, this latter comprises intrinsic impurities such as Fe^2+^/Fe^3+^ and Ni^2+^ ions, and other eventual traces of metallic pathogens. Furthermore, often, natural asbestos is characterized by the co-presence of several mineral phases, fiber dimensions of which are randomly distributed in width and in length. For these reasons, it is albeit challenging to precisely identify toxicity factors and to define the accurate role of each factor in the overall pathogenesis of asbestos. In this regard, the availability of synthetic asbestos fibers with accurate chemical composition and specific dimensions for in vitro screening tests would represent the perfect tool to correlate asbestos toxicity to its chemico-physical features. Herein, to palliate such drawbacks of natural asbestos, well-defined Ni-doped tremolite fibers were chemically synthesized in order to offer biologists adequate samples for testing the specific role of Ni^2+^ in asbestos toxicity. The experimental conditions (temperature, pressure, reaction time and water amount) were optimized to produce batches of asbestos fibers of the tremolite phase, with uniformly distributed shape and dimensions and a controlled content of Ni^2+^ metal ions.

## 1. Introduction

Asbestos is the general name employed to designate fibrous silicate minerals and includes serpentine (chrysotile) and the amphibole minerals: tremolite, actinolite, anthophyllite, amosite and crocidolite [1]. Owing to its desirable technological properties, asbestos has been exploited since ancient times as construction constituent and in the materials industry [2]. Unfortunately, it is now well documented and renown that asbestos can induce lung cancer, malignant mesothelioma and other asbestos-related diseases in exposed individuals [3]. All types of asbestos possess some degree of toxicity, but it would appear that asbestos amphiboles are considered the more dangerous ones [4]. Accumulated data suggest that different factors may be involved in the asbestos-related diseases such as dimensions of the fibers, chemical characteristics, surface reactivity and biopersistence [3,5], but the exact mechanisms by which cell damages are provoked still remains a crucial unanswered question. 

Nevertheless, several investigations have pointed out that asbestos toxicity may be related to the production of reactive oxygen species (ROS) [6]. The capability of these fibrous minerals to enhance ROS depends mainly on two features of the fibers: chemical composition and dimension.

**Figure 1:**
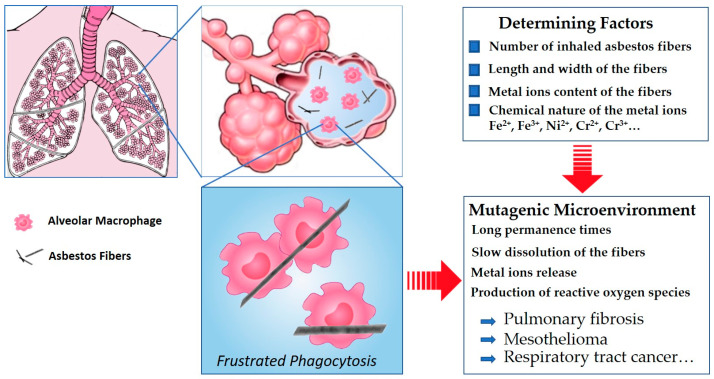
A scheme of the asbestos pathogenicity, showing the frustrated phagocytosis process.

Regarding the chemical composition, iron (Fe^2+^/Fe^3+^) is present in all varieties of natural asbestos either as a true component of the crystalline structure or as substituted/inserted cations. This metal seems to have a central role in asbestos toxicity [7,8,9]. In addition to iron, other elements in trace amounts such as Ni^2+^ and Cr^2+^/Cr^3+^ are taken into consideration as indicators of asbestos pathogenicity, although the specific role they play is not yet fully unveiled [10]. Consequently, asbestos fibers mainly act as complex crystal-chemical reservoirs [11,12,13] that may release potential toxic metal ions into the lung cellular environment during their permanence and slow dissolution [14]. A recent review by Kuroda [13] highlights the importance of frustrated phagocytosis occurring when alveolar macrophages attempt to remove the inhaled long fibers from the lung’s alveoli, thus creating a microenvironment prone to induce mutagenesis (Figure 1).

Figure 1 illustrates the first steps of the pathogenicity generated by the inhalation of asbestos fibers, showing the main determining factors involved in the instauration of the mutagenic microenvironment. A more complete view of the induced cellular responses in the lung after inhalation of asbestos fibers and a comprehensive description of the mechanisms of carcinogenesis by asbestos were recently provided by Mossman and Gualtieri [15], to which interested readers are referred.

So far, in an attempt to identify all the pathological mechanisms involved, in vitro studies have been performed using natural asbestos. In particular, tremolite asbestos was employed to correlate the toxicity of the mineral with both its chemical composition and morphology [16]. However, since the fibers of tremolite asbestos naturally vary in size and chemical composition and they are also often containing additional diverse mineral phases, an accurate distinction of factors of toxicity was still rather difficult to reach, especially in identifying the precise role of each factor in the pathogenesis of asbestos tremolite. Following this research line, with the aim of making distinction between the chemical composition vs. the fiber dimensions, synthetic tremolitic amphibole fibers Ca_2_Mg_5_Si_8_O_22_(OH)_2_ (end member) were synthesized under hydrothermal conditions [17] and an in vitro pilot study was realized to probe the functional and structural cell damages caused by exposure to natural and synthetic end-member tremolite asbestos fibers [18]. This study demonstrated that synthetic fibers exerted a more direct cytotoxic effect than the natural ones. However, after contact with natural tremolite fibers, the survival of damaged cells expressing high level of vascular endothelial growth factor was also detected, showing that further studies are still required to fully understand the noxiousness of asbestos amphiboles that is also most likely linked to its intrinsic chemical composition and structure.

Tremolite Ca_2_Mg_5_Si_8_O_22_(OH)_2_ belongs to a calcic amphibole group and its structure consists of two principal elements, both of which extend in the c-direction: (i) double chains of corner-sharing T(1) and T(2) tetrahedra occupied by Si^4+^, and (ii) strips of edge-sharing M(1), M(2) and M(3) octahedra occupied by Mg^2+^. At the junction of the strip of octahedra and the double-chain of tetrahedra is the M(4) site, which is occupied by Ca^2+^ [19]. Natural tremolite samples often have compositions that differ slightly from its minor elements. In fact, a considerable amount of toxic minor (i.e., Fe_tot_ as Fe^2+^-Fe^3+^) and trace elements (i.e., Ni^2+^, Mn^2+^, Co^2+^/Co^3+^) was determined to be allocated among the *M*(1,2,3) sites [20,21,22]. In this light, a more suitable method to correlate the toxicity of the asbestos to its chemical composition and/or morphology would be to perform in vitro screening tests by using several sets of fibers of homogeneous and controlled size and specific chemical composition. For these reasons and also based on our previous report on the growth of Ni-doped chrysotile asbestos fibers [23], we decided, in the current study, to focus our efforts on synthesizing Ni-doped tremolite asbestos fibers with a fine control of both their morphology, mineral phases and chemical composition. The Ni-doped tremolite fibers have been produced through hydrothermal synthesis from granular quartz and magnesium, calcium and nickel oxides used as starting raw materials. Among the metal ions known to be hazardous to human health, Ni^2+^ is considered the most hazardous one for the damages it causes to DNA [24,25]. Furthermore, due to its ability to generate ROS [26,27,28], Ni^2+^ can cause a variety of lethal diseases, such as pulmonary fibrosis and respiratory tract cancer [29,30]. In addition, carcinogenesis among nickel-processing workers has also been associated to the possible cofactor effect of inhalation of asbestos-containing dust [31]. It is noteworthy that a recent report assessed the cytotoxicity and intracellular dissolution of nickel nanowires suggesting a synergetic toxicity between the aspect ratio of the nanowires and the amount of dissolved Ni^2+^ ions [31]. Indeed, regarding the dimension of asbestos fibers (i.e., diameter D), several authors suggest that very thin fibers (D < 0.25 µm) may have greater carcinogenic potential than wider ones [8]. These reports therefore justify the need to access Ni-doped tremolite fibers with controlled chemical composition and size in order to help clarify the etiology of the cytotoxic outcome of asbestos.

For all these reasons, particular attention has been paid herein to correlate the experimental conditions used during the synthesis with the chemical and morphological/size features of these synthetic Ni-doped tremolite fibers. The synthesis was based on the crystallization of the phases under high temperatures and pressure. Despite the relatively high costs of the equipment for the hydrothermal synthesis, the possibility to synthesize large crystals of high quality and precise chemical composition still renders this technique far more beneficial for such a goal. Furthermore, through hydrothermal synthesis, it is possible to obtain the Ni-doped tremolite by adjusting the processing conditions, including temperature, pressure or concentration of starting materials, all parameters that can influence chemical composition, crystal phases and fiber dimension, and that can all be tuned in a systematic manner. The synthesis was carried out in stainless steel cylinder autoclaves, which can withstand high temperatures and pressures for a long time [32,33]. PXRD, EPMA, SEM/EDS, TEM/EDS and µ-R were used to characterize starting materials and the Ni-doped tremolite samples obtained.

## 2. Materials and Methods

### 2.1. Hydrothermal Synthesis of Ni-Doped Tremolite

**Figure 2:**
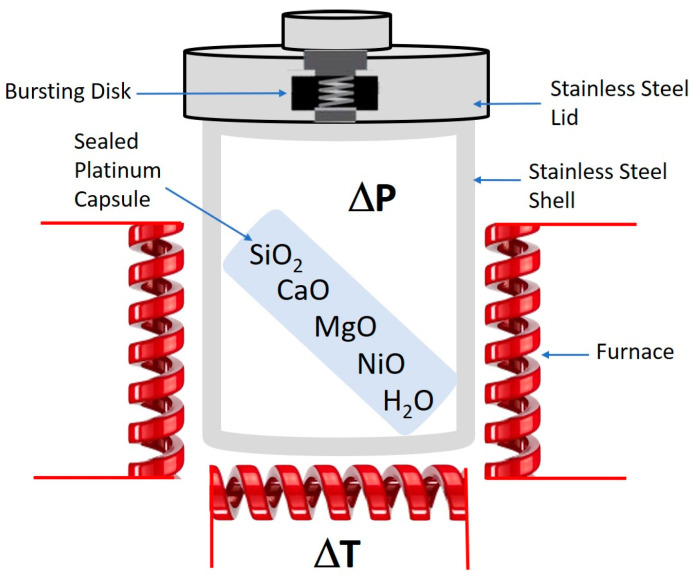
A scheme of the experimental process.

Ni-doped tremolite fibers were grown in externally heated pressure vessels (Figure 2). The following starting materials were used: granular quartz (SiO_2_; CarloErba reagent, code No. 364011), MgO (Carlo Erba reagent, code n. 459586), NiO nickel oxide (Sigma-Aldrich reagent, code No. 1313-99-1) and CaO. This latter one was obtained through decarboxylation by heating CaCO_3_ powder for 24 h (Carlo Erba reagent, code n. 433185) to 900 °C in order to remove CO_2_.

To increase the reactivity between starting materials, quartz and magnesium oxide were pre-treated as follows: granular quartz was converted into cristobalite by heating the powdered SiO_2_ to 1400 °C; MgO powder was converted into periclase under heating to 900 °C, while CaO and NiO were heated to 110 °C for several hours in order to insure their anhydrous form since both oxides are highly hygroscopic materials. About 60 mg of finely (0.177 mesh) powdered mixture of a molar composition of starting materials in accordance with an ideal Ca_2_(Mg_4_Ni_1_)Si_8_O_22_(OH)_2_ tremolite form, plus distilled water (pH = 7) were put within a sealed platinum capsule (0.11 cm^3^ in volume) and placed inside the externally heated pressure vessel [17]. Note that the amount of added distilled water was also modulated throughout the various runs according to Table 1. 

Several runs were carried out with reaction temperature ranging from 780 to 830 °C, under pressure from 1.1 to 2.4 kbar; and the reaction time was varied from 4 to 16 days. Experimental conditions of all runs are summarized in Table 1. Note that each run has been performed in triplicate to unsure reproducibility of the data. For each replicate, no significant variations in composition or fiber dimension were observed. 

Temperature was continuously monitored by two chromel–alumel thermocouples placed at the hottest position of the vessel. Internal pressure inside the externally heated vessel was supplied through a hydrostatic circuit and continuously monitored using a Nova Swiss transducer system. Pressure was transmitted to the sample contained in a sealed platinum capsule, through a hole in the top cover lid. 

After several days (between 4 and 16 days) during which the capsules were maintained at the same temperature and pressure, the vessel was removed from the furnace and, at run pressure, was initially quenched by applying a jet of air to the bottom of the vessel and successively the vessel was immerged into a water bath for the final cooling (cooling rate of ca. 300 °/min). Note that the growth conditions of Ni-doped tremolite have been chosen according to previous results [17,34]. Furthermore, the experimental conditions (temperature, pressure, reaction time and starting mixtures) chosen for the synthesis of end-member asbestos amphiboles aimed to (i) avoid the formation of additional phases as those reported in literature, e.g., [17,34], (ii) avoid the departure of composition of synthetic tremolite from its ideal stoichiometry. 

### 2.2. Characterization of Ni-Doped Tremolite Fibers

The run products were initially inspected by stereo binocular microscope (SBM) performed using an Askania, GSZ 2T, fitted with a digital camera (Fuji X-E2, magnification from 4 to 40×). The images of the Ni-doped tremolite were generated using the “focus stacking” technique. This method consists in taking a sufficient number of equally spaced photographs to spam the entire area to be kept in focus. This became necessary since as the shooting magnification increases, part of the object that remains in focus decreases. About one hundred photos were taken for a single image of Ni-doped tremolite fibers. The collected images were combined in Affinity Photo software and treated in order to obtain the best possible framing, but also to insure the removal of merging artefacts. Fiber length was also measured using a phase contrast optical microscope (MOCF) Askania, RML 5 using 40 and 10× magnification. 

Powder X-Ray diffraction (PXRD) was performed using a Bruker D8 Advance X-ray diffractometer working at 40 kV and 40 mA. The diffractometer is equipped with a copper tube and curved graphite monochromator. Scans were collected in a 2θ range of 3–66°, with a 3 s/step counting time of and a 0.02° step interval. EVA software (DIFFRACplus EVA) was used to identify the mineral phases and the collected reflection peaks were compared with the PDF2 reference patterns. 

Scanning electron microscopy (SEM) was performed using a ZEISS CrossBeam 350 Ultra High Resolution (UHR-SEM) equipped with a Spectrometer EDS—EDAX OCTANE Elite Plus—Silicon drift type.

To check the morphology of the samples, transmission electron microscopy (TEM) was carried out with Jeol JEM 1400 Plus (Tokyo, Japan) under 120 kV, equipped with a double tilt holder. In situ energy dispersive X-ray spectrometer (EDS) of the Jeol company was employed for microanalyses. Prior to investigations, all samples were sonicated in isopropyl alcohol. Three drops of the obtained suspension were deposited on a Formvar carbon-coated copper grid. In order to describe the width of the tremolite, several TEM micrographs were recorded and 1600 single fibers measured (200 for each run). Measurements on tremolite fibers were carried out using OPTIKA PROView image analysis software. Microchemical analysis of the samples was carried out using an Electron Probe Micro Analysis (EPMA) JEOL-JXA 8230 coupled with a Spectrometer EDS–JEOL EX-94310FaL1Q—Silicon drift type. The EDS analyses were carried out according to the following operating conditions: 11 mm working distance; 10 nA probe current; 15 keV HV; 30 s live time; and 40° take off. For each sample, tremolite crystals were selected using binocular microscopy in order to rule out the possibility of contamination of other mineral impurities (Table 1). The final chemical composition is the mean value of 4 spot analyses carried out on various tremolite crystals.

Sample TN8 was further investigated also with Micro-Raman Spectroscopy (µR-S). Micro-Raman analyses were effectuated using a Thermo Fisher DXR Raman microscope (Waltham, MA, USA), equipped with the OMNICxi Raman Imaging software 1.0, a 10× objective, a 900 ln/mm grating (full width at half maximum, FWHM), and an electron multiplying charge-coupled device (EMCCD). The 532 nm line (solid state laser) was employed at a 1.8 to 7 mW incident power output. We selected the spectral region from 1200 to 200 cm^−1^ as the area containing the lattice vibrational modes characteristic for amphiboles [35]. 

## 3. Results and Discussion

### 3.1. PXRD Characterization

**Figure 3:**
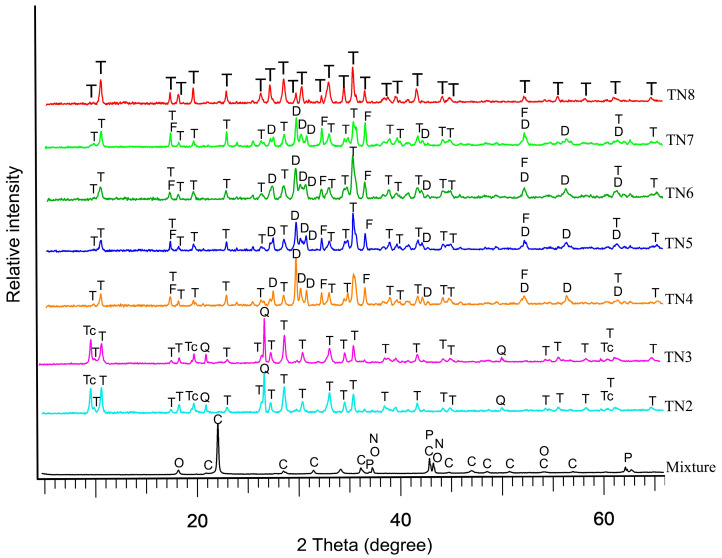
PXRD pattern from mixture and products. T = tremolite (JCPDS card 44-1402); C = cristobalite (JCPDS card 39-1425); P = periclase (JCPDS card 45-0946); O = calcium oxide (JCPDS card 44-1418); N = nickel oxide (JCPDS card 47-1049); Tc = talc (JCPDS card 29-1493); Q = quartz (JCPDS card 5-0490); D = diopside (JCPDS card 19-0239); F = forsterite (JCPDS card 34-0189). Peaks were assigned according to the literature.

In order to evaluate the best conditions to obtain monomineralic, abundant and long Ni-doped tremolite fibers, several attempts were carried out (Table 1). In the initial attempts, we worked in the presence of excess aqueous solution with respect to the required stoichiometric H_2_O amount hosted in tremolite (i.e., 2%) [21], and as we expected, the main phase that was formed was the talc doped with nickel. The choice to work in excess of H_2_O during alteration lies in previous results on synthesized tremolite [17,36,37]. PXRD patterns show that with the decrease in the pressure from 1.5 to 1.1 or 1.3 kbar (run TN4-TN6, Table 1), Ni-doped tremolite decreases in abundancy, as it can be clearly seen by the evident decrease in the tremolite peak intensity of the (110) reflection (Table 1 and Figure 3). 

However, an increase in pressure up to 1.7 kbar improved the hydrothermal synthesis. In particular, as pressure increases, the 110 reflection peak of tremolite clearly appears tighter and more intense as it can be seen in the TN7 diffractogram compared to the diffractograms of TN4, TN5, and TN6 samples (Figure 3). Moreover, a lower pressure, down to 1.1 kbar (Run TN4, TN5, TN6; Table 1), causes a reduction in Ni-doped tremolite abundance and an increase in Ni-doped diopside; this fact was clearly deduced by the intensity and the shape of (-2 2 1) reflection at 29.911 (2*θ*) of diopside. Finally, Ni-doped tremolite as single phase (Figure 3 and Figure S1, Supplementary Materials) was obtained after 16 days of reaction at 2.4 kbar and 800 °C using 8% of water reactant. It is noteworthy that in all runs, we obtained fibers identified as Ni-doped tremolite (Table 1) and unreacted starting materials were never observed. Ni-doped tremolite (run TN8) lattice parameters were calculated via refinement of the PXRD reflections (5° < 2θ < 60°) using Topas software. The refined obtained values are *a* = 9.8055(3) Å, *b* = 18.0741 (9) Å, *c* = 5.2791(4) Å, *β* = 104.6765 (7)°, to be compared with the lattice parameters extrapolated by Pacella et al. [16] for Maryland tremolite, *a* = 9.8523 Å, *b* = 18.0766 Å, *c* = 5.2812 Å, *β* = 104.7476°. On the whole, the unit cell volume for Ni-doped tremolite was 905.0607 Å^3^, thus slightly smaller than the unit cell volume of Maryland tremolite (V = 909.5757 Å^3^) used as a comparison. The amount of nickel dopant and the different ionic ratios of magnesium (0.72 Å) substituted by nickel (0.69 Å) in the synthesized tremolite reduces the cell dimensions below those of Ni-free tremolite. As reported in the literature, Ni^2+^ was incorporated in the tremolite substitutes for Mg^2+^ in the octahedral sites [1,38], its behavior therefore being similar to those observed in the synthesis of Ni-doped enstatite and Ni-doped chrysotile [23,39] so that the variation in cell dimensions is the function of the composition of the octahedral strip, which is affected by cation size.

### 3.2. SBM/MOCF/SEM/TEM/EDS Characterization

**Figure 4:**
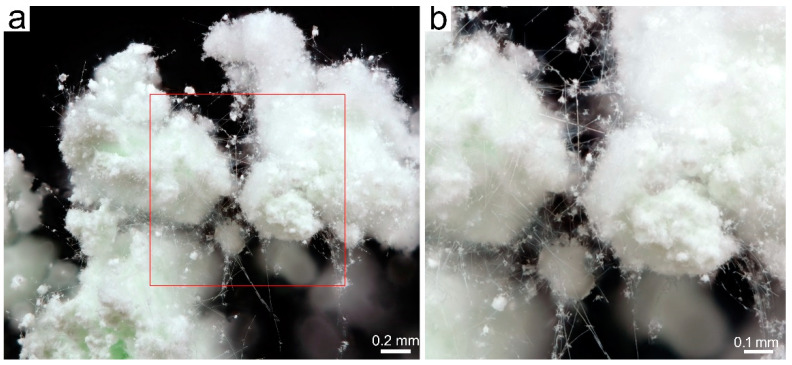
(**a**) Stereoscopic binocular microscope image of white fibers of Ni-doped tremolite run TN8; (**b**) enlargement of the red square of Figure (**a**).

The tremolite fibers were visible to the SBM in all runs, such as wads of fibers protruding the mass of the synthesized product released from the platinum capsules. Under SBM, as it can be observed in Figure 4a, the tremolite fibers curled up in bundles are white in color. Single curved fibers are clearly visible; they tend to split longitudinally (Figure 4b, and Figures S2 and S4, Supplementary Materials). The longest fibers of more than 2 mm in length were observed in the TN8 sample, whereas the length of the tremolite fibers of the other trials was shorter (Table 1) as detected by SBM, MOCF and SEM (Figures S3 and S4, Supplementary Materials). 

**Figure 5:**
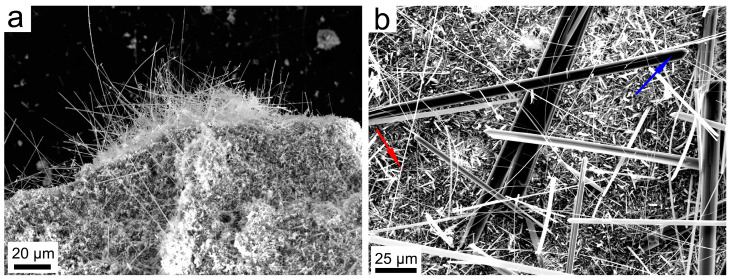
(**a**) Secondary electron SEM images of Ni-doped tremolite fibers run TN8 (800 °C, 2.4 kbar, 16 day); (**b**) note the curvature of the thin Ni-doped tremolite fibers (red arrow) and wider Ni-doped tremolite fibers (blue arrow).

**Figure 6:**
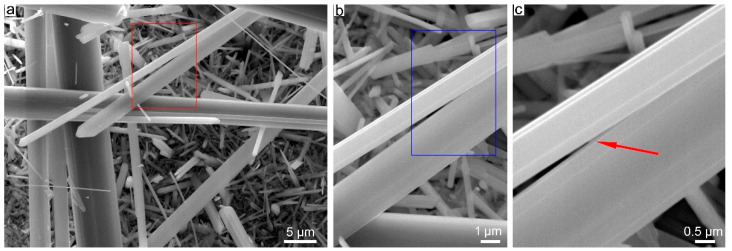
Secondary electron SEM images. (**a**) Longitudinal splitting into thinner fibrils from larger fiber of Ni-doped tremolite (red rectangle) run TN8 (800 °C, 2.4 kbar, 16 day); (**b**) enlargement of the red rectangle of Figure (**a**); (**c**) enlargement of the blue rectangle of Figure (**b**), note the fiber splitting indicated by the red arrow.

Under SEM, at low magnification (Figure 5a), it can be observed that the Ni-doped tremolite displays a fibrous morphology, and crystallizes as long bundles of fibers, several micrometers long. The presence of fibers of different width is observed at higher magnification (Figure 5b); the thinnest and longest fibers often appear curved (Figure 5b). Wider Ni-doped tremolite fibers appear rigid, although in some cases, the longitudinal splitting of larger fibers into thinner fibrils is evident (Figure 6). In Figure 6a, for example, the characteristic monoclinic prismatic morphology of Ni-doped tremolite with a cleavage parallel to the fiber axis can be observed. 

**Figure 7:**
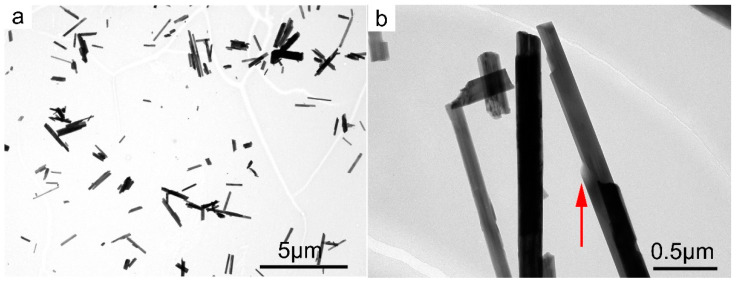
(**a**) TEM micrographs of Ni-doped tremolite run TN8 (800 °C, 2.4 kbar, 16 day); (**b**) fibers with different widths. Note structural defects along their elongation direction; the red arrow indicates edges at 120°.

Deeper morphological/microstructural observations on the single Ni-doped tremolite fibers were conducted by TEM equipped with EDS for microanalysis. Specifically, for the determination of the width, one of the main important characteristics which plays a significative role in asbestos toxicity, hundreds of TEM micrographs were recorded and at least 1400 single Ni-doped tremolite fibers were measured. Moreover, special attention was paid in the identification of other eventual synthesized products if present (Table 1). It is noteworthy that the high-resolution TEM images of run products showed only Ni-doped tremolite crystals (Figure 7a) in the run TN8, while in all the other runs the presence of accessory phases that were identified through PXRD was also confirmed (Table 1). 

**Figure 8:**
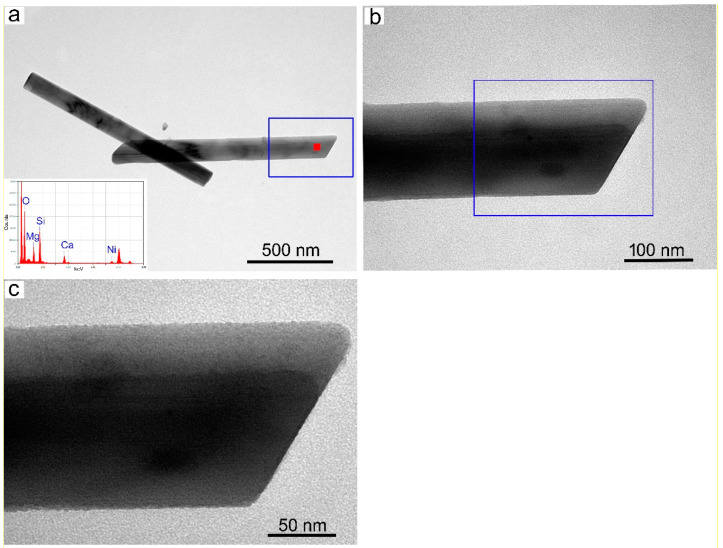
(**a**) TEM micrograph of single Ni-doped tremolite fibers with the relative EDS point analysis (red square); (**b**) zoom of the blue rectangle of Figure (**a**); (**c**) zoom of the blue rectangle of Figure (**b**).

Single Ni-doped tremolite can be observed with strain-shaped morphologies of parallel sides and regular termination (Figure 8) and flaking steps are observed at about 120° (Figure 7b). It is excluded that these are cleavage fragments of bladed prismatic tremolite since in that case the crystals would have had irregular sides and blunt edges [37]. However, at higher magnification, some fibers clearly showed structural defects (i.e., chain multiplicity faults—CMFs) elongated according to the direction of elongation, a feature commonly documented in the literature [1,37,40,41,42,43]. 

Under TEM, at low magnification, the single fibers appeared thin with of different width. As previously determined in other synthetic pure tremolite (end-member) [1] in which the size of the Ni-free tremolite fibers obtained covered a large width range, 0.1–15 mm [30,31,42,44,45], Ni-doped tremolite fibers also show variation in width (Table 1). The results as calculated by measuring 200 single fibers width for each run indicate 0.20 μm as average value ranging from 0.15 to 0.29 μm. 

**Figure 9:**
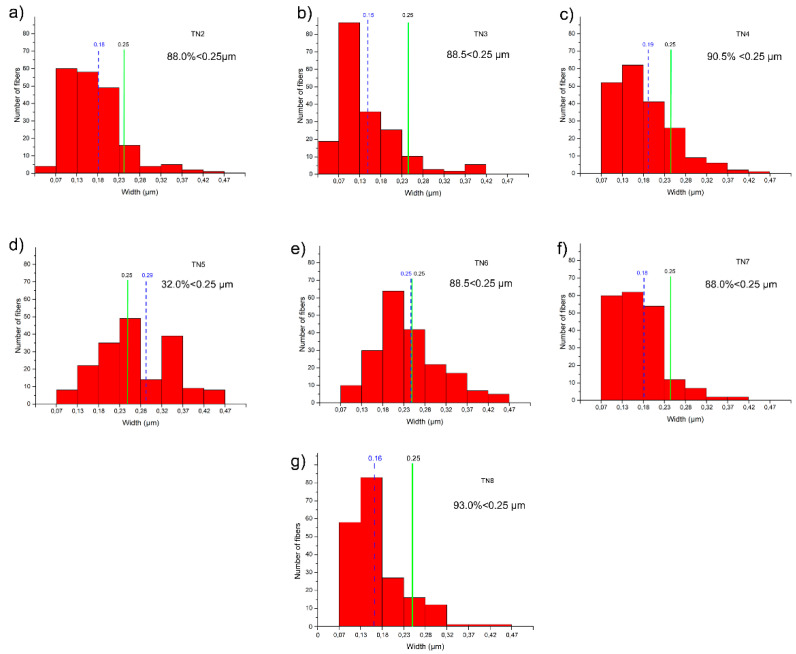
Width distribution of Ni-doped tremolite fibers; (**a**) run TN2 (88.0% < 0.25 µm); (**b**) run TN3 (88.5 < 0.25 µm); (**c**) run TN4 (90.5% < 0.25 µm); (**d**) run TN5 (32.0% < 0.25 µm); (**e**) run TN6 (88.5 < 0.25 µm); (**f**) run TN7 (88.0% < 0.25 µm) and (**g**) run TN8 (93.0% < 0.25 µm), as calculated by measuring 200 single fibers for each run. The blue line shows average value. The green line at 0.25 μm can be considered as a thickness threshold; hence, based on Stanton’s hypothesis these fibers may be considered potentially carcinogenic.

A distribution of the average width with the number of measurements for all runs is reported in Figure 9. Except the fibers of the run TN5, most of the fibers are less than 0.25 μm wide (Figure 9) which is considered as a width threshold under which, based on Stanton’s hypothesis [8], these fibers may be considered potentially carcinogenic. Indeed, TN5 which was effectuated under low temperature (780 °C), relatively low pressure (1.3 kbar) and an average amount of water (8 mL) is characterized by a higher number of fibers of wider width above 0.25 μm (68%). Decreasing the amount of water by a half greatly decreases the width of the fibers, as observed for TN6, for which only ca. 12% of the fibers remain with a width above 0.25 μm. Instead, increasing both temperature and pressure tends to decrease the width of the resulting fibers, the pressure having a higher role on this matter. Optimal conditions were therefore reached for TN8 (800 °C, 2.4 kbar, 8 mL of H_2_O) presenting more than 93% of the fibers with width under 0.25 μm. It is noteworthy that the duration of the experiment has no effect on the overall width of the fibers (TN2 vs. TN3), and only the quantity of isolated product is affected by long reaction time. Moreover, the Ni-doped tremolite fibers were generally >5 µm long (Table 1). This has to be taken into consideration since fibers >5 µm cannot be fully engulfed by macrophages (frustrated phagocytosis, see Figure 1) and can remain inside the lung for a longer time, triggering adverse effects and chronic inflammation [46]. Moreover, these sizes fall in the range of respirable fibers, defined by WHO (1986) [47] as fibers with a width ≤ 3 μm, length ≥ 5 μm, and length/width ratio ≥ 3:1. Due to the large ratio of overlap of the entangled fibers, the accurate measurement count of the high length fibers was not a feasible task. Indeed, to distinguish between asbestos fibers and non-asbestos particles, some authors [48,49] suggested to consider only the crystal width parameter. In this view, a width ≤ 1 µm was considered a possible criterion for distinguishing asbestiform amphibole from non-asbestos particles. The variations observed in size of Ni-doped tremolite fibers were attributed to the different experimental conditions employed for their synthesis.

### 3.3. EPMA Characterization

EPMA analysis of several tremolite fibers, as expected, revealed the presence of Si, Mg, Ca and Ni, while no other elements (as impurities) were detected on the fibers. Chemical data on individual samples (Table 2) are consistent with the chemical composition of tremolite according to the excel data sheet built by Tindle and Webb 1994 [50]. The atoms for Ni-doped tremolite, calculated on the basis of 23 oxygen atoms per formula unit, showed that the amount of Ni ranges from 0.4 to 1.2 a.p.f.u. (Table 2). Except for run TN8, as expected, the dopant amount of the obtained tremolite was altered by the formation of other phases, thus producing tremolite fibers with composition far from the reagent ratio compositions of the starting mixture. 

Only in the monophase run TN8 did Ni-doped tremolite fibers show direct correlations between the quantity of Ni^2+^ content and the reagent molar ratios in the starting precursor mixture. 

Although synthesized tremolite (TN8) shows a slightly lower Ca/Mg value (0.36) of the ideal products of the starting mixtures (0.5), this Mg excess in synthetic tremolite is considered as Mg-cummingtonite Mg_7_Si_8_O_22_(OH)_2_ component depending on the thermodynamic growth conditions [36,51]. Indeed, it is worth mentioning that, on the basis of the numerous experimental researches carried out on synthesized tremolite, synthetic tremolite products tend to be cummingtonite-tremolite solid solutions with variable (7–3%) Mg amount [17,37,41,44,45,52]. 

### 3.4. µ-Raman Characterization

**Figure 10:**
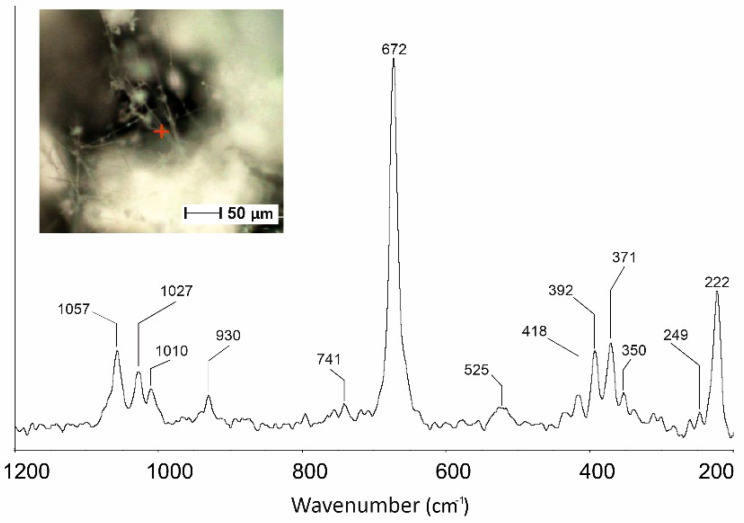
Micro-Raman spectrum of the Ni-doped tremolite run TN8 (800 °C, 2.4 kbar, 16 days).

Fibers from run TN8, in which tremolite was detected as the only phase, was also characterized by µ-Raman spectroscopy. The micro-Raman analyses of Ni-doped tremolite have shown four intense bands at 1057, 1027, 930 and 672 cm^−1^, and some weaker bands at 1010 and 741 cm^−1^ (Figure 10). The bands at 1057, 1027 and 1010 cm^−1^ are ascribed to the antisymmetric stretching vibrations (ν_as_) of Si-O-Si groups; the band at 930 cm^−1^ is produced by symmetric stretching vibrations of O-Si-O- linkage in agreement with the literature data [17,35,53,54]. The band at 1010 cm^−1^ observed in the Raman spectra is characteristic of synthetic tremolite [17]. The bands at 741 and 672 cm^−1^ are produced by the symmetric stretching vibrations (ν_as_) of the Si-O-Si groups [17]. The 672 cm^−1^ band characterized by the highest intensity shows an asymmetric shape compared to that collected on the natural samples which is usually symmetric [35,54,55]. Asymmetric shape is a characteristic of synthetic tremolite and appears due to the occurrence of pyribole lamellae. The bands at 525 may be ascribed to Si_4_O_11_ deformation modes. Different bands are detected in the spectral region of 650–300 cm^−1^ (418, 392, 371 and 350 cm^−1^); in this part of the spectrum lie the Mg–OH and Ni–OH vibrations, Si–O–Si bending motions and vibrational modes of the hydroxyls groups [17,55].

**Figure 11:**
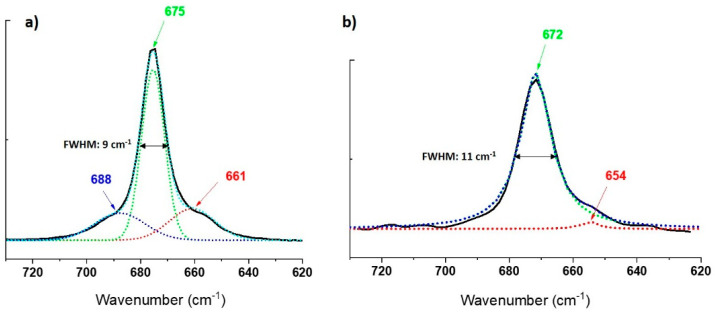
(**a**) Gaussian deconvolution of the asymmetric band at 675 cm^−1^ on the Raman spectrum of pure tremolite, three components were identified at 688, 675 and 661 cm^−1^ (dash lines); black line experimental band; (**b**) Lorentzian deconvolution of the asymmetric band at 672 cm^−1^ on the Raman spectrum of Ni-doped tremolite (run TN8), two components were identified at 672 and 654 cm^−1^; black line experimental band. FWHM = full width at half maximum.

The bands in the spectral region ranging from 300 to 200 cm^−1^ are produced by O-H-O motifs [17]. In this region, two bands are detected, at 249 and 222 cm^−1^. When the spectrum of TN8 fibers is compared with the Raman spectrum from pure synthetic tremolite [23], shifts in some vibrational modes can be identified (Figure 10). As matter of fact, bands at 1062, 1030, 932, 675, 531, 397, 372, 350, 252 and 225 cm^−1^ produced by Si-Ob-Si, M-O lattice mode (M = Ca, Mg, Ni) and O-H-O groups are now shifted toward lower wave-numbers (i.e., 1057, 1027, 930, 672, 525, 392, 371, 350, 249 e 222 cm^−1^), while the Raman bands at 1010, 741 and 418 cm^−1^ do not show any significant shift. The observed shifts toward lower frequencies may be attributed to the presence of Ni^2+^ cations within the octahedral sites in the synthesized tremolite which affect lattice parameters and cell volume dimensions. It is noteworthy that, as Mg^2+^ is substituted by Ni^2+^, the Raman bands that are shifted also broaden (Figure 11). 

Looking into the details at the main band centered at 672 cm^−1^, the latter changes its width (FWHM) from 9 cm^−1^ (675 cm^−1^) for pure tremolite Ca_2_Mg_5_Si_8_O_22_(OH)_2_ free of Ni^2+^ [17] to 11 cm^−1^ for Ni doped tremolite. Moreover, while the Raman band in pure tremolite adopts a gaussian profile as it is expected for solid samples, and in particular can be deconvoluted by three gaussian functions at 661, 675 and 688 cm^−1^, respectively (Figure 11a), the corresponding band at 672 cm^−1^ for the Ni-doped tremolite (sample TN8) can only be fitted by Lorentzian functions. In that case, two components can be identified at 654 and 672 cm^−1^ (Figure 11b). The contribution of the third expected band is probably too weak to be clearly identified. In this case, the necessity to use Lorentzian functions, which are usually employed for the deconvolution of Raman bands of liquids or gas, must therefore be related to the more “chaotic environment” owing to the introduction of the Ni^2+^ atoms into the crystal structure of the tremolite. 

## 4. Conclusions

In the present paper, we provide synthetic samples of Ni-doped tremolite characterized by a well-defined morphology, controlled dimensions and precise chemical composition to be used as standards to probe the interaction with controlled systems (simulated lung biofluids) in order to assess the degree of hazardousness of these asbestos fibers. The possibility to have Ni-doped tremolite with increasing Ni-content ranging between 0.4 and 1.2 a.p.f.u. could help to obtain in vitro data, not currently available, on the significance of Ni-cation in the induction of lung cell alterations. Based on the measured size, the most synthesized Ni-doped tremolite fibers may be considered potentially carcinogenic. 

The monomineralic Ni-doped tremolite fibers (run TN8) obtained at 800 °C, 2.4 kbar and reaction times of 16 days, free from extraneous mineral phases, could be more suitable to start the in vitro tests. Furthermore, considering the shift in the Raman bands registered for the first time in Ni–doped tremolite, this may represent quite a valuable resource in the framework of the enlargement of Raman databases. As a matter of fact, the data presented here may be relevant to compare Micro-Raman bands of other Ni-doped tremolite naturally occurring in other parts of the world. 

The data accumulated in this research can support the biological researchers in in vitro experiments in order to obtain unambiguous results on the role of nickel etiology of the cytotoxic outcome of tremolite asbestos not yet obtained from studies conducted through the use of natural tremolite fibers.

## Figures and Tables

**Table 1:** Experimental conditions and product list of synthesis for each run, in the order of decreasing abundance, as detected by PXRD, SEM/EDS, EDS/EPMA. TEM/EDS and µ-R used only for run TN8. Ni-Tr = Ni-doped tremolite; Ni-Tlc = Ni-doped talc; Ni-Fo = Ni-doped forsterite; Ni-Di = Ni-doped diposide. Full width at half-maximum (FWHM). The statistical error for FWHM was estimated at ~10%, whereas for peak intensity it was <5%.

Run	T (°C)	P (kbar)	t (Day)	H_2_O (wt.%)	Mineral Phases	Ni-Tr FWHM (110) (Δ°2θ)	Ni-Tr Peak Intensity (110) (cps)	Maximum * Length (µm)	Average ** Width (µm)
TN2	800	1.5	4	15	Ni-Tlc > Ni-Tr > Qtz	0.230	845	20	0.16
TN3	800	1.5	8	15	Ni-Tlc > Ni-Tr > Qtz	0.229	751	30	0.15
TN4	830	1.1	7	2	Ni-Di > Ni-Tr >> Ni-Fo	0.236	395	50	0.19
TN5	780	1.3	10	8	Ni-Tr > Ni-Di > Ni-Fo	0.260	336	200	0.30
TN6	780	1.3	10	4	Ni-Tr > Ni-Di > Ni-Fo	0.344	289	2500	0.25
TN7	800	1.7	7	10	Ni-Tr > Ni-Di > Ni-Fo	0.227	447	1500	0.18
TN8	800	2.4	16	8	Ni-Tr	0.267	717	5000	0.16

* as detected by SBM, MOCF and SEM/EDS (Supplementary materials Figures S1–S4). ** Average calculated on 200 individual fibers for each run (Supplementary materials Excel S6).

**Table 2:** Representative analyses of Ni-doped tremolite, normalized oxide weight percent and cation number (a.p.f.u.) calculated on the basis of 23 oxygens, from four EPMA analyses on tremolite crystals.

Oxides	TN2	TN3	TN4	TN5	TN6	TN7	TN8
SiO_2_	58.2 (0.5)	56.2 (0.8)	55.7 (1.1)	56.9 (0.4)	55.2 (0.3)	56.3 (0.4)	58.8 (0.4)
MgO	22.1 (0.4)	18.4 (1.3)	19.9 (1.4)	21.3 (0.9)	21.9 (0.1)	21.4 (0.9)	21.5 (0.4)
CaO	14.9 (0.5)	14.3 (1.5)	15.1 (0.6)	17.6 (1.2)	17.1 (0.1)	18.7 (1.5)	10.4 (0.6)
NiO	4.8 (0.1)	11.0 (0.9)	9.4 (2.0)	4.2 (0.2)	5.9 (0.5)	3.6 (0.4)	9.1 (0.8)
**CATIONS calculated on the basis of 23 oxygens**							
Si	7.9	7.9	7.8	7.8	7.7	7.7	8.0
Mg	4.5	3.8	4.1	4.4	4.5	4.4	4.4
Ca	2.2	2.2	2.2	2.5	2.5	2.8	1.6
Ni	0.5	1.2	1.1	0.5	0.6	0.4	1.0

## Data Availability

The data presented in this study are available on request from the corresponding author. The data are not publicly available due to ongoing research activities.

## Supplementary Materials

The following supporting information can be downloaded at: https://www.mdpi.com/article/10.3390/nano13081303/s1, Figure S1. Conventional Rietveld plots of Ni-doped tremolite run TN8; Figure S2. Optical image of Ni-doped tremolite run TN8; Figure S3. SEM image Ni-doped tremolite (run TN3-TN7); Figure S4. MOCF images of Ni-doped tremolite run TN8; Figure S5. TEM images of Ni-doped tremolite run TN8; Excel S6. measured widths by TEM image.
