# Supplementary material for: Synthesis of Ni-Doped Tremolite Fibers to Help Clarify the Aetiology of the Cytotoxic Outcome of Asbestos
Source: Nanomaterials (Basel). 2023 Apr 7;13(8):1303. doi: 10.3390/nano13081303 (PMC10142192; doi:10.3390/nano13081303)
Supplement: Supplementary file 1 [file nanomaterials-13-01303-s001.zip › nanomaterials-2295442-supplementary.pdf]

nanomaterials

Supplementary Materials

Synthesis of Ni-Doped Tremolite Fibers to Help Clarify the Aetiology of the Cytotoxic Outcome of Asbestos

Andrea Bloise [1,2,*], Eugenia Giorno [3], Domenico Miriello [1] and Nicolas Godbert [3]

1 Department of Biology, Ecology and Earth Sciences, University of Calabria, 87036 Rende (CS), Italy;

2 University Museum System—SiMU, section of Mineralogy and Petrography, University of Calabria, 87036 Arcavacata di Rende (CS), Italy

3 MAT-INLab Laboratory of Inorganic Molecular Materials, Department of Chemistry and Chemical Technologies, University of Calabria 87036 Rende (CS), Italy

* Correspondence: andrea.bloise@unical.it

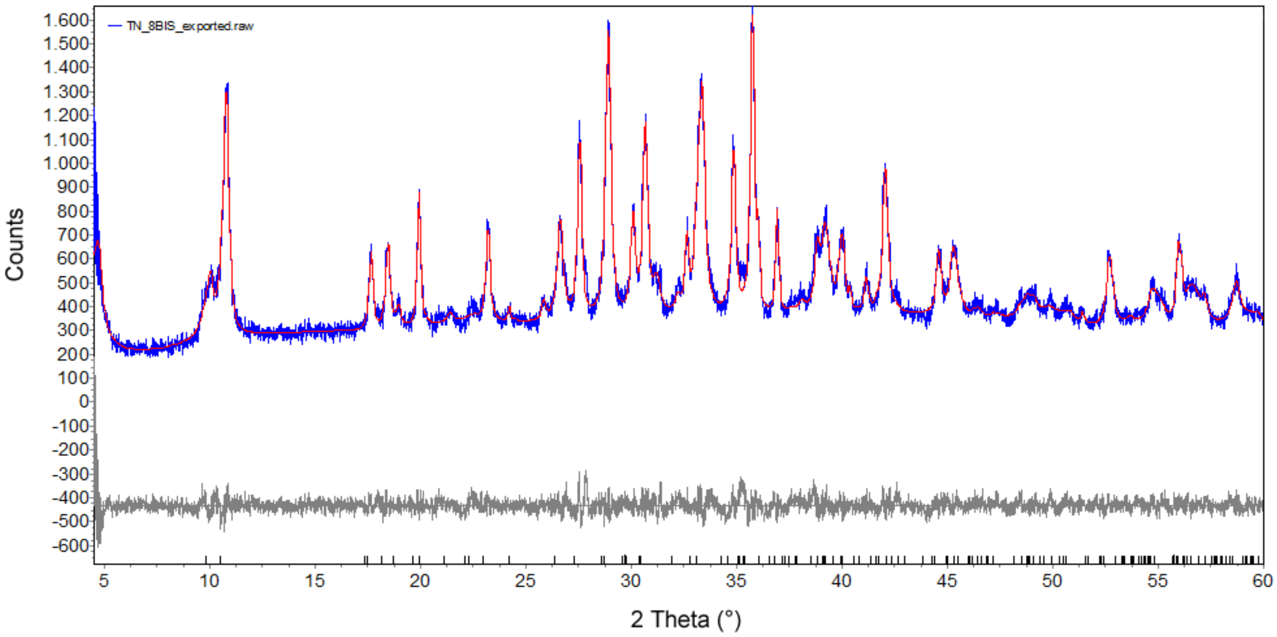

Figure S1. Conventional Rietveld plots of Ni-doped tremolite run TN8. Blue dots: experimental pattern; continuous red line: calculated pattern; continuous grey line: difference plot. Black vertical bars represent the position of Bragg reflectionof tremolite. Agreement factor Rwp = 6.7.

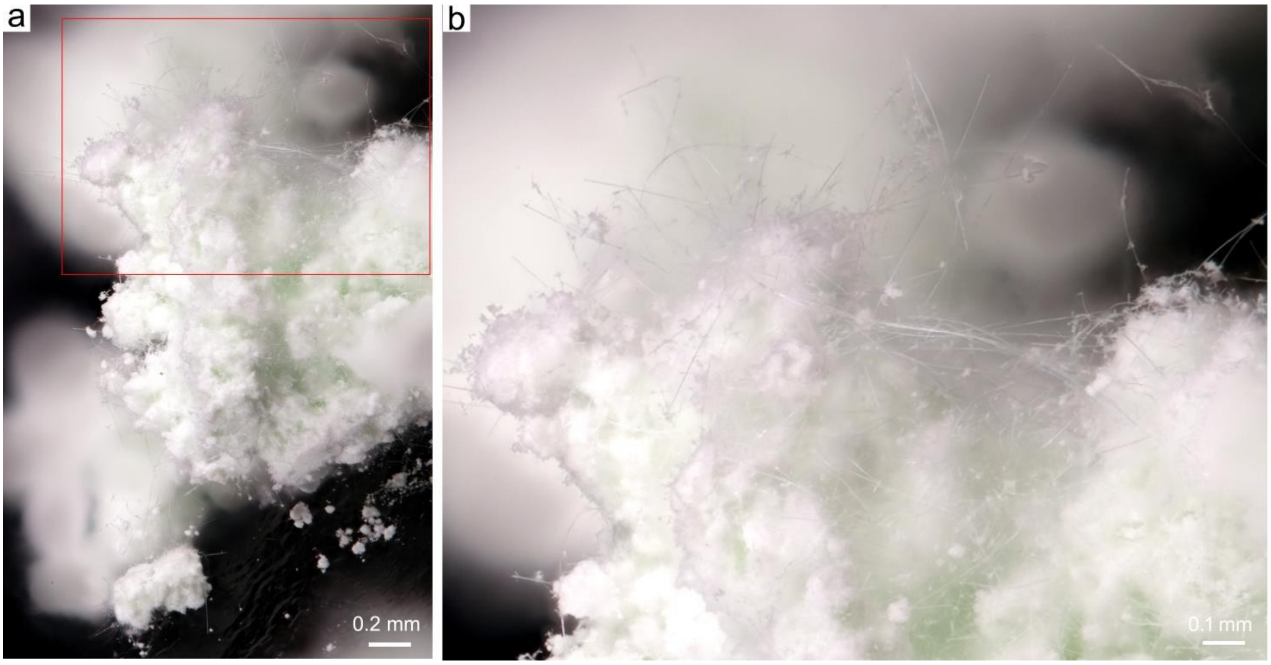

Figure S2. (**a**) optical image of white fibers of Ni-doped tremolite run TN8, (**b**) enlargement of the red square of figure a. Measurements on Ni-doped tremolite fibers were carried out using OPTIKA PROView image analysis software.

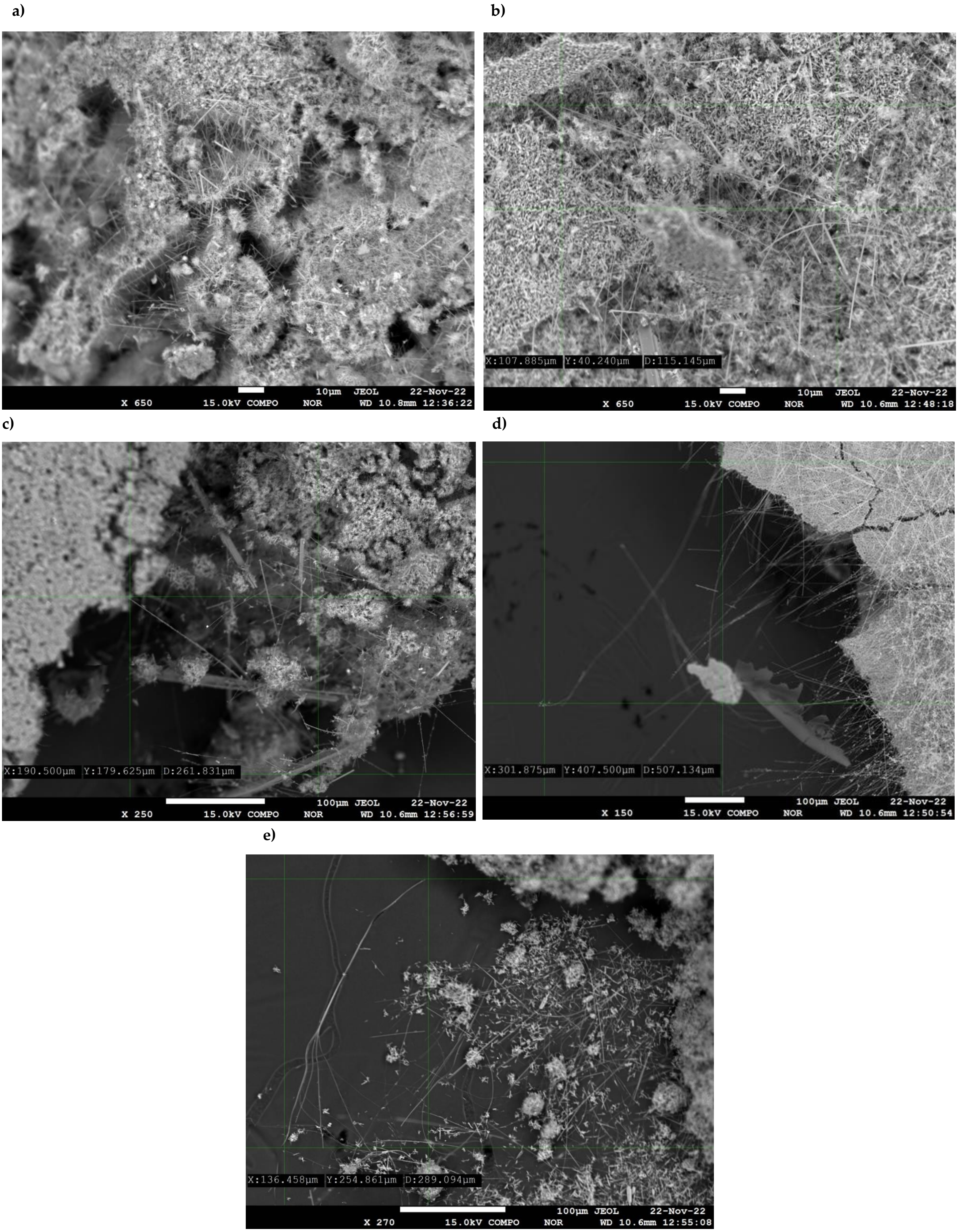

Figure S3. SEM images of Ni-doped tremolite. Selected representative images only a) run TN3, b) .run TN4 , c) run TN5, d) run TN6, e) TN7

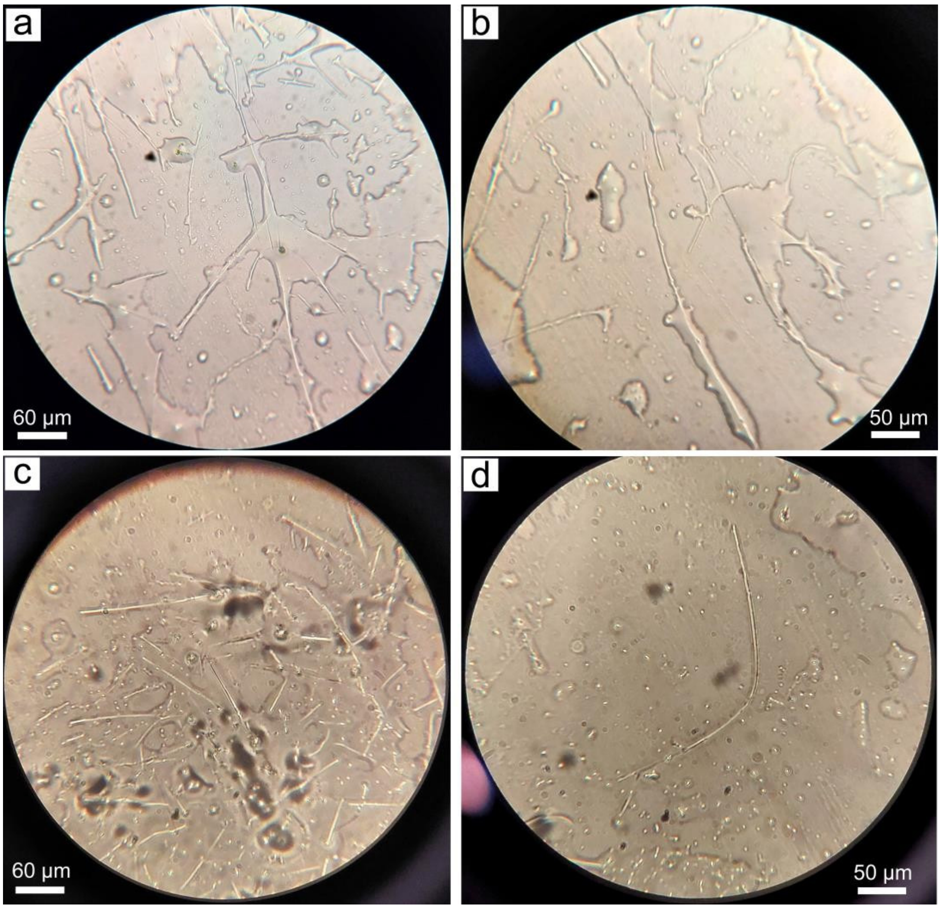

Figure S4. Selected MOCF images of the synthesized runs (**a-b**) Ni-doped tremolite fibers in liquid with refractive index of 1.615 (without phase contrast vision), (**c-d**) Ni-doped tremolite in liquid with refractive index of 1.615 (phase contrast vision).

a) b)

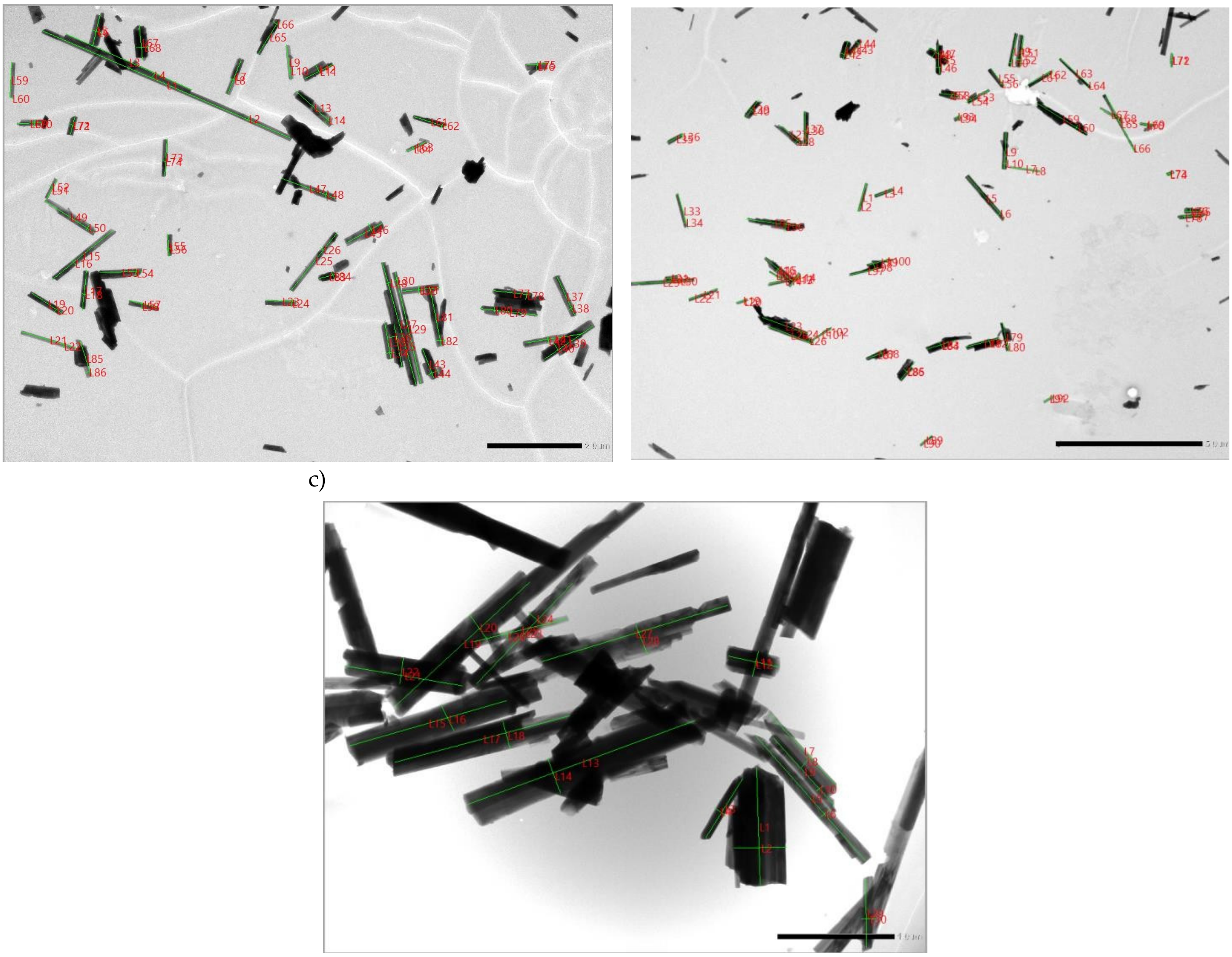

Figure S5. Example of TEM images of Ni-doped tremolite run TN8. Low magnification a) and b). High magnification c) Note, only width measurements have been used in this paper (see excel supplementary materials). Measurements on Ni-doped tremolite fibers were carried out using OPTIKA PROView image analysis software. Please note, the magnification at which the widths were measured is not the one shown in the following images (show as an example only).

Excel S6. measured widths by TEM images for all runs.

	TN2			TN3	
measure	coordinates	width (µm)	measure	coordinates	width (µm)
L1	(21.91, 16.82)	0,46	L1	(30.98, 36.86)	0,39
L2	(22.58, 17.86)	0,24	L2	(25.14, 35.83)	0,23
L3	(22.30, 19.18)	0,35	L3	(34.18, 30.25)	0,24
L4	(25.78, 18.04)	0,24	L4	(48.49, 44.83)	0,22
L5	(16.16, 18.11)	0,53	L5	(43.94, 50.77)	0,28
L6	(16.94, 26.32)	0,38	L6	(53.84, 48.79)	0,41
L7	(27.62, 23.52)	0,13	L7	(66.12, 46.34)	0,4
L8	(29.13, 20.09)	0,15	L8	(67.21, 44.10)	0,28
L9	(31.06, 16.42)	0,12	L9	(69.97, 44.93)	0,23
L10	(38.58, 13.46)	0,15	L10	(70.69, 43.90)	0,34
L11	(39.79, 14.82)	0,15	L11	(72.14, 42.07)	0,2
L12	(39.71, 18.26)	0,34	L12	(72.46, 41.00)	0,29
L13	(41.03, 19.77)	0,18	L13	(73.09, 39.63)	0,16
L14	(49.65, 17.40)	0,12	L14	(68.92, 38.75)	0,41
L15	(48.12, 15.23)	0,2	L15	(68.72, 39.96)	0,14
L16	(46.74, 12.48)	0,15	L16	(65.82, 38.59)	0,29
L17	(34.81, 11.73)	0,25	L17	(66.47, 36.17)	0,27
L18	(34.93, 13.36)	0,12	L18	(66.53, 35.33)	0,18
L19	(34.21, 13.21)	0,17	L19	(65.84, 34.54)	0,09
L20	(32.40, 13.53)	0,27	L20	(61.84, 31.70)	0,2
L21	(31.44, 11.66)	0,24	L21	(61.64, 31.42)	0,09
L22	(23.34, 11.16)	0,15	L22	(57.71, 29.75)	0,04
L23	(22.77, 10.28)	0,21	L23	(58.43, 28.62)	0,08
L24	(15.78, 13.86)	0,27	L24	(65.40, 31.66)	0,17
L25	(8.09, 32.40)	0,29	L25	(66.45, 32.95)	0,11
L26	(6.74, 32.29)	0,19	L26	(69.67, 32.11)	0,33
L27	(8.58, 30.87)	0,21	L27	(73.91, 31.36)	0,2
L28	(9.08, 30.00)	0,18	L28	(73.39, 30.44)	0,2
L29	(16.50, 33.86)	0,17	L29	(41.83, 42.75)	0,13
L30	(17.70, 29.82)	0,12	L30	(41.81, 42.23)	0,09
L31	(13.96, 27.58)	0,15	L31	(42.23, 41.91)	0,06
L32	(10.21, 24.75)	0,17	L32	(42.11, 41.35)	0,06
L33	(9.99, 23.08)	0,23	L33	(42.41, 40.90)	0,11
L34	(10.79, 22.05)	0,13	L34	(41.69, 40.94)	0,08
L35	(5.40, 23.89)	0,11	L35	(42.17, 39.55)	0,16
L36	(5.32, 21.64)	0,19	L36	(40.02, 40.84)	0,16
L37	(8.97, 17.45)	0,24	L37	(39.63, 41.28)	0,09
L38	(26.66, 26.85)	0,16	L38	(39.23, 42.21)	0,11
L39	(33.36, 29.77)	0,11	L39	(48.01, 45.53)	0,29
L40	(35.94, 29.02)	0,25	L40	(40.08, 47.00)	0,06

L41	(35.78, 32.73)	0,21	L41	(38.73, 45.05)	0,13
L42	(34.88, 32.32)	0,21	L42	(36.61, 42.75)	0,09
L43	(47.82, 32.38)	0,33	L43	(35.67, 41.47)	0,2
L44	(48.67, 28.91)	0,24	L44	(33.88, 41.85)	0,2
L45	(49.47, 26.64)	0,29	L45	(33.01, 41.99)	0,13
L46	(53.78, 25.07)	0,21	L46	(33.39, 43.32)	0,11
L47	(50.43, 24.05)	0,18	L47	(34.68, 43.74)	0,2
L48	(53.14, 21.26)	0,13	L48	(34.08, 44.34)	0,13
L49	(53.74, 20.50)	0,09	L49	(34.50, 45.41)	0,14
L50	(52.51, 16.42)	0,12	L50	(35.93, 47.73)	0,24
L51	(51.45, 16.73)	0,25	L51	(38.14, 47.30)	0,09
L52	(51.96, 13.81)	0,12	L52	(39.45, 48.89)	0,14
L53	(51.57, 13.37)	0,11	L53	(42.21, 49.09)	0,08
L54	(53.84, 11.96)	0,17	L54	(45.83, 48.45)	0,11
L55	(54.50, 13.48)	0,15	L55	(47.32, 48.31)	0,13
L56	(55.10, 15.72)	0,21	L56	(49.30, 48.35)	0,13
L57	(55.85, 17.86)	0,19	L57	(51.35, 47.87)	0,12
L58	(53.74, 22.05)	0,09	L58	(48.71, 49.38)	0,17
L59	(51.89, 22.10)	0,09	L59	(35.85, 54.79)	0,21
L60	(50.07, 19.91)	0,27	L60	(35.10, 54.81)	0,08
L61	(45.63, 20.18)	0,21	L61	(35.71, 54.07)	0,06
L62	(46.04, 18.71)	0,23	L62	(37.56, 52.82)	0,08
L63	(45.97, 17.92)	0,19	L63	(37.98, 51.73)	0,13
L64	(47.64, 38.77)	0,28	L64	(41.06, 52.80)	0,21
L65	(50.59, 37.65)	0,21	L65	(43.58, 54.03)	0,13
L66	(53.96, 36.20)	0,21	L66	(42.83, 54.45)	0,09
L67	(50.66, 33.72)	0,12	L67	(33.92, 45.71)	0,04
L68	(45.70, 35.01)	0,21	L68	(33.31, 45.07)	0,11
L69	(43.58, 38.71)	0,26	L69	(32.57, 42.96)	0,04
L70	(39.97, 39.38)	0,18	L70	(31.64, 44.10)	0,12
L71	(39.21, 39.30)	0,13	L71	(31.50, 44.53)	0,06
L72	(37.39, 39.03)	0,13	L72	(30.90, 45.27)	0,09
L73	(36.22, 39.40)	0,15	L73	(29.51, 44.44)	0,09
L74	(34.40, 38.17)	0,07	L74	(43.34, 44.20)	0,09
L75	(33.90, 37.79)	0,03	L75	(42.19, 44.28)	0,14
L76	(31.07, 35.60)	0,11	L76	(46.01, 44.46)	0,06
L77	(31.96, 39.35)	0,21	L77	(47.65, 44.24)	0,08
L78	(34.19, 40.98)	0,15	L78	(39.96, 55.60)	0,12
L79	(36.70, 40.78)	0,15	L79	(41.57, 55.48)	0,11
L80	(39.88, 40.78)	0,18	L80	(45.19, 55.64)	0,08
L81	(42.60, 40.91)	0,16	L81	(47.69, 54.75)	0,09
L82	(49.18, 36.20)	0,21	L82	(47.77, 54.23)	0,04
L83	(51.61, 29.79)	0,17	L83	(49.24, 54.89)	0,11
L84	(54.11, 27.96)	0,15	L84	(50.75, 54.53)	0,08
L85	(24.77, 34.68)	0,15	L85	(50.42, 53.26)	0,04
L86	(24.81, 37.27)	0,13	L86	(48.93, 52.19)	0,08
L87	(24.21, 38.42)	0,12	L87	(48.01, 52.36)	0,09
L88	(24.52, 39.50)	0,13	L88	(47.75, 52.40)	0,06
L89	(26.48, 40.50)	0,13	L89	(46.70, 53.38)	0,08
L90	(28.62, 40.56)	0,19	L90	(46.38, 52.94)	0,11

L91	(29.77, 32.24)	0,15	L91	(45.87, 52.44)	0,09
L92	(28.48, 29.78)	0,19	L92	(7.55, 50.70)	0,09
L93	(29.35, 28.62)	0,12	L93	(8.45, 49.42)	0,38
L94	(35.72, 3.96)	0,24	L94	(12.58, 49.64)	0,39
L95	(40.81, 5.82)	0,22	L95	(12.04, 48.79)	0,16
L96	(46.58, 6.47)	0,21	L96	(14.65, 48.61)	0,2
L97	(51.96, 6.09)	0,23	L97	(16.65, 46.70)	0,16
L98	(50.28, 8.39)	0,15	L98	(19.38, 46.04)	0,13
L99	(49.35, 7.16)	0,21	L99	(18.68, 50.42)	0,2
L100	(55.03, 4.12)	0,09	L100	(17.71, 50.68)	0,16
L101	(46.01, 1.94)	0,19	L101	(16.51, 51.41)	0,13
L102	(31.88, 2.42)	0,09	L102	(16.28, 51.95)	0,16
L103	(27.68, 2.38)	0,17	L103	(14.65, 52.68)	0,23
L104	(27.76, 4.60)	0,15	L104	(13.87, 53.00)	0,27
L105	(24.03, 3.23)	0,21	L105	(10.31, 54.09)	0,12
L106	(20.91, 3.26)	0,19	L106	(9.50, 54.23)	0,27
L107	(44.08, 0.62)	0,18	L107	(8.13, 51.73)	0,13
L108	(45.94, 1.14)	0,15	L108	(7.43, 51.79)	0,24
L109	(50.75, 2.39)	0,09	L109	(7.07, 52.23)	0,22
L110	(20.98, 10.07)	0,22	L110	(5.64, 50.38)	0,2
L111	(20.18, 12.96)	0,21	L111	(6.34, 47.52)	0,13
L112	(16.95, 11.39)	0,15	L112	(6.40, 46.70)	0,16
L113	(35.51, 10.78)	0,16	L113	(9.24, 46.20)	0,2
L114	(37.43, 9.18)	0,2	L114	(10.69, 45.95)	0,22
L115	(37.43, 8.20)	0,09	L115	(11.59, 46.44)	0,13
L116	(42.99, 14.00)	0,12	L116	(12.54, 46.48)	0,17
L117	(21.74, 39.94)	0,16	L117	(13.06, 45.65)	0,13
L118	(15.26, 39.19)	0,15	L118	(3.97, 41.79)	0,16
L119	(12.07, 39.40)	0,17	L119	(5.98, 41.65)	0,14
L120	(7.24, 35.76)	0,19	L120	(9.12, 42.15)	0,17
L121	(4.75, 34.85)	0,2	L121	(11.11, 42.71)	0,06
L122	(19.71, 35.21)	0,24	L122	(13.65, 42.89)	0,2
L123	(21.47, 35.98)	0,19	L123	(12.12, 44.28)	0,18
L124	(22.46, 37.68)	0,06	L124	(11.74, 44.42)	0,13
L125	(20.35, 37.64)	0,16	L125	(4.77, 46.22)	0,11
L126	(25.57, 36.82)	0,15	L126	(1.71, 43.82)	0,09
L127	(23.62, 33.21)	0,09	L127	(1.97, 42.17)	0,14
L128	(25.06, 31.30)	0,15	L128	(1.57, 45.39)	0,2
L129	(23.05, 30.48)	0,09	L129	(3.97, 46.82)	0,18
L130	(27.01, 28.33)	0,18	L130	(4.99, 48.65)	0,2
L131	(32.30, 24.63)	0,21	L131	(5.94, 49.22)	0,06
L132	(46.39, 24.72)	0,12	L132	(2.33, 39.90)	0,12
L133	(46.80, 23.74)	0,18	L133	(3.48, 39.86)	0,12
L134	(43.80, 23.14)	0,23	L134	(5.13, 40.38)	0,08
L135	(41.51, 23.23)	0,16	L135	(6.86, 40.88)	0,13
L136	(40.37, 23.34)	0,11	L136	(9.56, 39.98)	0,2
L137	(34.13, 9.24)	0,24	L137	(14.21, 40.44)	0,06
L138	(33.93, 10.28)	0,34	L138	(15.52, 39.94)	0,12
L139	(32.35, 9.84)	0,12	L139	(17.47, 40.36)	0,13
L140	(29.55, 8.83)	0,12	L140	(19.81, 42.81)	0,09

L141	(26.66, 7.61)	0,21	L141	(20.45, 43.36)	0,2
L142	(32.48, 6.66)	0,23	L142	(21.30, 43.76)	0,24
L143	(31.88, 6.11)	0,09	L143	(20.75, 44.87)	0,08
L144	(33.93, 4.15)	0,09	L144	(20.05, 45.05)	0,13
L145	(36.66, 2.80)	0,21	L145	(16.93, 44.83)	0,18
L146	(46.13, 7.48)	0,21	L146	(22.58, 52.68)	0,14
L147	(48.45, 8.01)	0,12	L147	(23.39, 53.20)	0,13
L148	(53.24, 8.52)	0,17	L148	(24.64, 53.46)	0,11
L149	(55.87, 5.43)	0,13	L149	(25.77, 53.85)	0,14
L150	(55.88, 2.18)	0,21	L150	(26.31, 50.89)	0,16
L151	(3.94, 24.85)	0,17	L151	(22.28, 49.40)	0,14
L152	(5.88, 24.97)	0,15	L152	(21.48, 48.97)	0,2
L153	(2.96, 35.97)	0,21	L153	(20.51, 50.12)	0,18
L154	(2.17, 36.69)	0,12	L154	(19.97, 51.91)	0,14
L155	(2.23, 35.65)	0,15	L155	(20.11, 53.22)	0,08
L156	(1.80, 38.27)	0,09	L156	(19.79, 53.74)	0,11
L157	(3.97, 39.43)	0,27	L157	(19.50, 53.87)	0,06
L158	(6.98, 39.71)	0,12	L158	(19.38, 54.99)	0,13
L159	(8.81, 40.38)	0,21	L159	(19.48, 55.48)	0,18
L160	(12.96, 33.70)	0,23	L160	(20.77, 55.52)	0,09
L161	(10.69, 31.88)	0,23	L161	(27.56, 52.44)	0,13
L162	(22.17, 27.65)	0,21	L162	(29.31, 52.54)	0,14
L163	(21.52, 29.74)	0,33	L163	(29.13, 51.91)	0,08
L164	(30.48, 34.87)	0,19	L164	(29.69, 51.41)	0,24
L165	(38.05, 34.78)	0,32	L165	(30.94, 50.87)	0,09
L166	(40.57, 32.79)	0,09	L166	(25.20, 50.75)	0,11
L167	(41.28, 30.88)	0,12	L167	(25.18, 48.45)	0,21
L168	(41.17, 29.60)	0,07	L168	(26.33, 48.45)	0,14
L169	(34.12, 18.17)	0,15	L169	(26.31, 54.73)	0,11
L170	(33.04, 17.80)	0,29	L170	(27.05, 55.62)	0,06
L171	(35.04, 16.64)	0,15	L171	(25.40, 55.37)	0,08
L172	(14.31, 31.51)	0,12	L172	(23.09, 55.44)	0,11
L173	(20.70, 33.90)	0,12	L173	(23.37, 54.05)	0,16
L174	(22.87, 32.38)	0,13	L174	(23.27, 53.74)	0,09
L175	(28.72, 35.73)	0,09	L175	(28.60, 54.83)	0,06
L176	(27.76, 30.63)	0,21	L176	(27.84, 54.29)	0,09
L177	(27.46, 30.00)	0,07	L177	(30.72, 54.89)	0,11
L178	(50.53, 11.03)	0,19	L178	(35.43, 53.42)	0,12
L179	(49.91, 12.48)	0,11	L179	(33.74, 52.80)	0,09
L180	(49.35, 12.04)	0,03	L180	(34.24, 48.99)	0,06
L181	(54.52, 10.38)	0,06	L181	(35.63, 49.72)	0,12
L182	(51.99, 7.05)	0,16	L182	(21.70, 47.30)	0,08
L183	(52.58, 4.46)	0,18	L183	(19.22, 48.95)	0,17
L184	(42.83, 2.29)	0,12	L184	(18.10, 54.69)	0,14
L185	(38.87, 1.29)	0,11	L185	(17.51, 55.68)	0,2
L186	(37.79, 0.38)	0,4	L186	(14.15, 54.07)	0,09
L187	(37.55, 1.91)	0,2	L187	(27.70, 41.93)	0,08
L188	(25.32, 0.44)	0,09	L188	(26.97, 41.93)	0,14
L189	(25.32, 1.29)	0,07	L189	(27.25, 43.66)	0,13
L190	(24.47, 1.58)	0,21	L190	(29.33, 42.98)	0,16

L191	(22.90, 1.47)	0,18	L191	(30.19, 40.86)	0,09
L192	(22.18, 0.72)	0,17	L192	(23.83, 46.54)	0,09
L193	(29.38, 1.41)	0,18	L193	(26.69, 46.08)	0,13
L194	(27.43, 5.13)	0,07	L194	(34.94, 47.34)	0,25
L195	(30.38, 6.26)	0,15	L195	(36.84, 45.71)	0,08
L196	(27.99, 10.41)	0,25	L196	(37.04, 43.96)	0,18
L197	(28.59, 11.38)	0,17	L197	(35.08, 33.58)	0,12
L198	(27.73, 12.02)	0,15	L198	(36.61, 33.56)	0,09
L199	(26.20, 12.13)	0,15	L199	(35.04, 37.92)	0,04
L200	(30.79, 13.84)	0,12	L82	(27.41, 22.22)	0,13
**Average**		**0,18**	**Average**		**0,15**

TN4

measure	coordinates	width (µm)
L1	(1.86, 20.25)	0,15
L2	(2.18, 18.24)	0,16
L3	(0.37, 17.17)	0,15
L4	(1.11, 16.24)	0,16
L5	(5.90, 18.13)	0,21
L6	(5.55, 18.24)	0,17
L7	(7.53, 17.34)	0,13
L8	(8.33, 16.82)	0,18
L9	(7.52, 13.38)	0,18
L10	(7.04, 9.68)	0,28
L11	(7.88, 9.90)	0,09
L12	(6.58, 9.09)	0,16
L13	(3.25, 8.12)	0,11
L14	(2.94, 8.03)	0,13
L15	(2.94, 7.25)	0,15
L16	(1.77, 6.87)	0,13
L17	(1.97, 6.56)	0,16
L18	(1.32, 7.26)	0,16
L19	(0.98, 7.29)	0,19
L20	(0.74, 7.73)	0,21
L21	(3.54, 4.83)	0,26
L22	(3.90, 4.45)	0,13
L23	(3.25, 3.99)	0,27
L24	(2.41, 1.80)	0,23
L25	(4.34, 0.48)	0,27
L26	(12.71, 2.32)	0,11
L27	(17.04, 1.32)	0,14
L28	(18.87, 0.95)	0,13
L29	(17.53, 2.65)	0,15
L30	(14.63, 3.05)	0,11
L31	(14.35, 3.24)	0,15
L32	(15.21, 8.43)	0,13
L33	(15.72, 8.93)	0,15
L34	(13.86, 9.16)	0,19
L35	(13.66, 8.76)	0,14
L36	(12.19, 8.03)	0,13
L37	(13.37, 9.79)	0,23
L38	(15.35, 11.70)	0,12
L39	(16.36, 11.65)	0,22
L40	(13.02, 11.92)	0,3

TN5

measure	coordinates	width (µm)
L1	(114.55, 53.38)	0,23
L2	(116.21, 51.49)	0,36
L3	(117.18, 50.40)	0,32
L4	(120.73, 49.60)	0,23
L5	(120.05, 49.94)	0,23
L6	(121.82, 48.91)	0,34
L7	(120.33, 46.39)	0,11
L8	(117.53, 46.62)	0,23
L9	(114.49, 48.63)	0,36
L10	(100.63, 26.58)	0,57
L11	(106.64, 29.44)	0,23
L12	(108.30, 26.00)	0,34
L13	(115.52, 28.29)	0,34
L14	(118.38, 27.61)	0,11
L15	(118.27, 36.08)	0,41
L16	(133.91, 37.46)	0,23
L17	(132.99, 37.86)	0,26
L18	(137.34, 38.66)	0,47
L19	(132.82, 41.81)	0,26
L20	(127.09, 42.55)	0,16
L21	(123.31, 44.22)	0,34
L22	(121.25, 45.07)	0,16
L23	(111.97, 41.18)	0,36
L24	(109.68, 39.92)	0,16
L25	(108.71, 40.38)	0,41
L26	(102.69, 34.14)	0,34
L27	(97.88, 30.36)	0,34
L28	(98.74, 32.99)	0,23
L29	(94.16, 48.45)	0,23
L30	(94.85, 43.41)	0,32
L31	(93.93, 44.22)	0,23
L32	(92.55, 52.18)	0,26
L33	(105.90, 55.78)	0,41
L34	(88.03, 54.01)	0,16
L35	(85.68, 49.20)	0,62
L36	(82.30, 43.59)	0,36
L37	(85.22, 41.75)	0,26
L38	(77.38, 53.38)	0,41
L39	(76.80, 54.07)	0,26
L40	(74.57, 53.78)	0,26

L41	(12.63, 11.37)	0,12	L41	(73.20, 53.55)	0,26
L42	(10.35, 11.54)	0,19	L42	(69.70, 53.89)	0,36
L43	(12.89, 12.81)	0,09	L43	(68.21, 54.81)	0,36
L44	(12.66, 13.82)	0,15	L44	(66.32, 56.01)	0,51
L45	(13.10, 14.60)	0,12	L45	(62.60, 58.42)	0,26
L46	(11.36, 12.36)	0,16	L46	(61.80, 58.71)	0,49
L47	(14.82, 14.17)	0,2	L47	(57.85, 59.56)	0,46
L48	(15.71, 14.31)	0,23	L48	(54.35, 59.97)	0,16
L49	(17.69, 9.69)	0,17	L49	(53.67, 60.37)	0,41
L50	(22.95, 10.07)	0,24	L50	(53.44, 61.05)	0,11
L51	(23.51, 10.75)	0,22	L51	(44.04, 58.82)	0,49
L52	(24.50, 11.70)	0,17	L52	(42.73, 61.17)	0,51
L53	(26.02, 12.10)	0,39	L53	(68.84, 75.72)	0,51
L54	(24.96, 12.12)	0,11	L54	(66.78, 77.09)	0,46
L55	(22.83, 11.28)	0,09	L55	(69.99, 79.44)	0,26
L56	(22.18, 9.99)	0,3	L56	(69.36, 80.36)	0,36
L57	(22.36, 9.57)	0,12	L57	(67.64, 82.53)	0,58
L58	(24.02, 8.69)	0,24	L58	(66.27, 82.76)	0,36
L59	(25.76, 8.25)	0,14	L59	(65.12, 83.10)	0,36
L60	(19.34, 9.15)	0,53	L60	(62.54, 83.10)	0,77
L61	(18.88, 8.99)	0,32	L61	(61.45, 83.39)	0,57
L62	(18.82, 8.66)	0,26	L62	(59.97, 82.93)	0,11
L63	(25.58, 4.03)	0,19	L63	(58.76, 85.57)	0,23
L64	(24.65, 4.81)	0,17	L64	(60.37, 85.28)	0,11
L65	(25.63, 5.35)	0,11	L65	(55.67, 86.48)	0,32
L66	(26.94, 3.69)	0,09	L66	(88.49, 73.48)	0,16
L67	(29.69, 2.61)	0,17	L67	(99.37, 68.96)	0,11
L68	(28.24, 3.58)	0,19	L68	(79.90, 69.59)	0,36
L69	(28.02, 3.36)	0,21	L69	(78.58, 72.85)	0,32
L70	(28.16, 2.61)	0,18	L70	(75.72, 75.14)	0,32
L71	(27.97, 1.46)	0,24	L71	(71.99, 73.14)	0,36
L72	(27.82, 0.87)	0,12	L72	(79.78, 66.67)	0,26
L73	(29.71, 1.17)	0,2	L73	(84.77, 65.64)	0,32
L74	(30.89, 1.85)	0,17	L74	(84.48, 66.27)	0,36
L75	(31.91, 1.81)	0,13	L75	(89.23, 63.23)	0,23
L76	(31.47, 1.62)	0,13	L76	(92.10, 62.77)	0,23
L77	(33.22, 1.29)	0,17	L77	(99.89, 62.43)	0,23
L78	(27.90, 5.45)	0,39	L78	(100.00, 63.63)	0,26
L79	(29.48, 5.08)	0,11	L79	(98.85, 62.89)	0,32
L80	(29.91, 5.27)	0,08	L80	(103.49, 61.57)	0,36
L81	(30.32, 5.53)	0,18	L81	(104.75, 60.37)	0,26
L82	(30.57, 5.76)	0,19	L82	(96.91, 56.93)	0,32
L83	(30.30, 9.40)	0,11	L83	(103.72, 55.38)	0,36
L84	(31.93, 10.25)	0,27	L84	(104.18, 52.12)	0,26
L85	(30.75, 10.69)	0,12	L85	(152.18, 47.71)	0,36
L86	(27.62, 10.40)	0,23	L86	(148.85, 48.34)	0,41
L87	(27.83, 11.43)	0,25	L87	(147.25, 44.67)	0,47
L88	(32.43, 8.53)	0,14	L88	(143.47, 43.76)	0,26
L89	(33.02, 8.77)	0,18	L89	(143.41, 41.70)	0,32
L90	(30.32, 7.94)	0,19	L90	(144.10, 40.78)	0,23

L91	(29.05, 7.68)	0,18	L91	(142.15, 48.74)	0,26
L92	(27.93, 8.04)	0,17	L92	(153.49, 51.72)	0,26
L93	(28.46, 8.42)	0,32	L93	(157.39, 52.29)	0,26
L94	(37.50, 8.97)	0,13	L94	(158.42, 51.72)	0,26
L95	(36.07, 8.43)	0,25	L95	(160.65, 51.09)	0,34
L96	(35.85, 6.46)	0,27	L96	(146.91, 49.77)	0,16
L97	(34.76, 4.98)	0,13	L97	(134.42, 50.80)	0,16
L98	(33.74, 4.78)	0,31	L98	(134.77, 47.65)	0,41
L99	(33.74, 7.32)	0,15	L99	(134.71, 46.39)	0
L100	(33.29, 6.99)	0,25	L100	(138.09, 45.59)	0,26
L101	(32.73, 6.73)	0,16	L101	(134.19, 55.44)	0,26
L102	(38.98, 7.73)	0,21	L102	(132.93, 55.50)	0,16
L103	(39.68, 8.07)	0,19	L103	(133.33, 58.42)	0,23
L104	(40.49, 8.18)	0,17	L104	(142.10, 59.28)	0,16
L105	(40.68, 7.25)	0,13	L105	(145.65, 59.28)	0,49
L106	(40.56, 6.41)	0,12	L106	(169.93, 49.03)	0,34
L107	(37.36, 4.88)	0,09	L107	(168.56, 49.08)	0,36
L108	(37.32, 5.13)	0,16	L108	(165.29, 48.22)	0,46
L109	(41.70, 5.99)	0,12	L109	(174.28, 45.19)	0,36
L110	(41.88, 5.18)	0,15	L110	(176.00, 48.80)	0,26
L111	(39.62, 11.97)	0,23	L111	(176.63, 52.81)	0,23
L112	(40.72, 12.13)	0,25	L112	(178.35, 53.38)	0,23
L113	(41.14, 12.82)	0,29	L113	(178.12, 51.60)	0,26
L114	(38.30, 12.81)	0,13	L114	(174.68, 55.04)	0,34
L115	(37.70, 12.75)	0,36	L115	(184.82, 58.65)	0,26
L116	(37.10, 12.70)	0,21	L116	(184.48, 57.33)	0,16
L117	(37.66, 11.53)	0,24	L117	(184.82, 56.99)	0,16
L118	(27.95, 17.70)	0,2	L118	(194.62, 54.35)	0,26
L119	(33.36, 15.48)	0,15	L119	(197.31, 55.15)	0,36
L120	(23.73, 17.68)	0,24	L120	(199.66, 56.59)	0,23
L121	(25.08, 18.33)	0,23	L121	(186.43, 61.80)	0,36
L122	(25.74, 18.77)	0,18	L122	(185.05, 61.28)	0,34
L123	(32.85, 16.95)	0,24	L123	(189.18, 63.69)	0,34
L124	(33.56, 17.90)	0,21	L124	(192.33, 64.38)	0,23
L125	(32.39, 18.61)	0,16	L125	(194.27, 63.34)	0,23
L126	(28.82, 16.13)	0,37	L126	(196.51, 63.46)	0,26
L127	(29.71, 15.16)	0,36	L127	(180.47, 63.75)	0,36
L128	(27.08, 15.56)	0,11	L128	(171.13, 57.96)	0,23
L129	(27.65, 15.09)	0,18	L129	(169.30, 58.25)	0,26
L130	(32.15, 14.52)	0,25	L130	(167.24, 58.08)	0,23
L131	(32.35, 13.76)	0,14	L131	(159.51, 56.19)	0,16
L132	(33.24, 14.23)	0,13	L132	(155.50, 65.06)	0,26
L133	(36.44, 16.96)	0,11	L133	(164.60, 63.40)	0,26
L134	(37.72, 16.82)	0,24	L134	(130.01, 55.50)	0,26
L135	(37.87, 17.44)	0,19	L135	(130.24, 54.47)	0,26
L136	(39.74, 17.65)	0,24	L136	(129.32, 50.86)	0,32
L137	(39.93, 14.40)	0,31	L137	(128.75, 49.89)	0,26
L138	(38.34, 14.31)	0,27	L138	(129.95, 49.08)	0,16
L139	(37.18, 14.41)	0,17	L139	(126.63, 53.78)	0,36
L140	(37.74, 22.45)	0,2	L140	(124.63, 54.18)	0,23

L141	(38.99, 23.58)	0,13	L141	(123.25, 54.93)	0,26
L142	(37.63, 23.83)	0,26	L142	(126.46, 57.90)	0,26
L143	(36.42, 23.37)	0,2	L143	(129.90, 59.34)	0,32
L144	(34.28, 22.53)	0,21	L144	(139.06, 56.13)	0,23
L145	(33.10, 23.05)	0,19	L145	(116.27, 58.99)	0,23
L146	(32.77, 23.30)	0,24	L146	(115.18, 58.48)	0,16
L147	(31.90, 28.11)	0,47	L147	(112.94, 59.91)	0,23
L148	(35.95, 28.59)	0,14	L148	(112.71, 49.31)	0,26
L149	(36.78, 29.05)	0,13	L149	(111.45, 49.94)	0,23
L150	(36.57, 29.45)	0,12	L150	(111.86, 47.02)	0,36
L151	(37.84, 28.35)	0,23	L151	(113.80, 44.73)	0,16
L152	(38.91, 27.28)	0,13	L152	(116.95, 39.18)	0,23
L153	(39.63, 26.21)	0,17	L153	(118.38, 40.09)	0,26
L154	(38.76, 25.79)	0,23	L154	(116.27, 40.84)	0,26
L155	(38.61, 25.51)	0,14	L155	(113.06, 37.29)	0,26
L156	(37.11, 25.74)	0,32	L156	(41.07, 76.06)	0,26
L157	(40.10, 25.25)	0,16	L157	(47.82, 84.25)	0,36
L158	(35.68, 25.14)	0,21	L158	(49.60, 84.59)	0,11
L159	(35.68, 23.63)	0,36	L159	(46.74, 88.43)	0,23
L160	(35.76, 20.81)	0,15	L160	(31.50, 99.43)	0,23
L161	(35.83, 21.24)	0,17	L161	(35.17, 98.74)	0,32
L162	(35.07, 20.37)	0,26	L162	(34.82, 102.18)	0,23
L163	(35.51, 20.14)	0,13	L163	(33.96, 104.41)	0,16
L164	(35.15, 19.50)	0,19	L164	(30.64, 104.30)	0,36
L165	(33.06, 20.85)	0,36	L165	(44.96, 100.00)	0,26
L166	(32.10, 20.41)	0,16	L166	(45.76, 97.37)	0,47
L167	(31.38, 21.91)	0,19	L167	(47.82, 96.56)	0,26
L168	(38.14, 19.04)	0,17	L168	(43.18, 95.59)	0,11
L169	(37.40, 18.85)	0,17	L169	(43.24, 94.27)	0,26
L170	(37.43, 18.26)	0,24	L170	(42.04, 92.90)	0,23
L171	(38.73, 18.94)	0,2	L171	(23.02, 82.53)	0,26
L172	(35.91, 17.59)	0,23	L172	(20.50, 81.96)	0,26
L173	(36.82, 16.08)	0,08	L173	(19.76, 83.10)	0,16
L174	(36.72, 15.41)	0,09	L174	(18.84, 101.37)	0,47
L175	(32.80, 15.76)	0,18	L175	(22.22, 102.41)	0,23
L176	(34.33, 9.52)	0,29	L176	(19.99, 104.64)	0,36
L177	(35.31, 9.95)	0,17	L177	(20.10, 105.27)	0,26
L178	(36.09, 9.72)	0,2	L178	(17.81, 105.78)	0,16
L179	(38.56, 3.95)	0,12	L179	(15.81, 105.38)	0,51
L180	(39.22, 2.32)	0,22	L180	(14.83, 105.10)	0,16
L181	(37.47, 0.87)	0,09	L181	(14.55, 103.95)	0,26
L182	(37.26, 0.95)	0,11	L182	(12.26, 102.86)	0,46
L183	(35.79, 2.63)	0,15	L183	(11.00, 102.46)	0,57
L184	(41.70, 3.38)	0,18	L184	(13.92, 109.79)	0,36
L185	(42.59, 3.28)	0,15	L185	(14.78, 109.05)	0,32
L186	(41.47, 2.15)	0,17	L186	(11.11, 107.62)	0,26
L187	(42.68, 0.97)	0,09	L187	(7.39, 111.28)	0,36
L188	(40.51, 3.56)	0,12	L188	(6.01, 110.08)	0,41
L189	(40.87, 5.09)	0,22	L189	(5.15, 110.42)	0,51
L190	(41.78, 9.53)	0,24	L190	(5.27, 113.92)	0,26

L191	(41.91, 9.86)	0,15
L192	(42.50, 9.80)	0,14
L193	(42.66, 10.66)	0,19
L194	(40.37, 9.92)	0,16
L195	(38.02, 8.60)	0,16
L196	(37.45, 8.45)	0,11
L197	(37.65, 8.30)	0,09
L198	(39.86, 7.55)	0,11
L199	(38.69, 5.88)	0,17
L200	(38.22, 5.88)	0,33
**Average**		**0,19**

L191	(2.81, 114.95)	0,36
L192	(2.81, 107.45)	0,26
L193	(2.23, 106.59)	0,16
L194	(4.93, 109.16)	0,23
L195	(29.27, 108.13)	0,62
L196	(27.95, 108.02)	0,32
L197	(43.81, 104.64)	0,16
L198	(43.41, 101.78)	0,26
L199	(42.44, 101.60)	0,41
L200	(43.07, 98.74)	0,23
**Average**		**0,29**

	TN6	
measure	coordinates	width (µm)
L1	(68.87, 51.89)	0,42
L2	(55.59, 63.50)	0,33
L3	(52.11, 65.80)	0,39
L4	(52.93, 64.21)	0,32
L5	(53.31, 63.47)	0,33
L6	(62.43, 65.44)	0,24
L7	(62.84, 64.43)	0,23
L8	(73.77, 63.64)	0,39
L9	(89.10, 67.41)	0,15
L10	(98.79, 67.63)	0,15
L11	(109.99, 48.14)	0,2
L12	(85.60, 6.00)	0,12
L13	(60.90, 9.78)	0,12
L14	(59.97, 11.36)	0,2
L15	(56.74, 9.45)	0,44
L16	(29.22, 2.03)	0,23
L17	(25.33, 41.46)	0,27
L18	(20.97, 43.81)	0,11
L19	(22.97, 45.40)	0,33
L20	(2.98, 47.86)	0,27
L21	(16.70, 69.22)	0,35
L22	(17.14, 70.32)	0,15
L23	(4.68, 70.12)	0,28
L24	(17.83, 4.46)	0,17
L25	(88.11, 38.53)	0,2
L26	(85.13, 38.66)	0,16
L27	(85.32, 38.12)	0,15
L28	(85.95, 35.98)	0,12
L29	(81.08, 42.55)	0,16
L30	(98.55, 42.93)	0,16
L31	(108.51, 40.69)	0,16
L32	(103.07, 37.57)	0,22
L33	(103.42, 37.87)	0,17
L34	(101.56, 35.82)	0,16
L35	(101.48, 37.70)	0,2
L36	(101.20, 39.07)	0,2
L37	(100.22, 39.95)	0,12
L38	(100.03, 41.76)	0,17
L39	(100.82, 42.36)	0,12
L40	(96.77, 41.37)	0,2

	TN7	
measure	coordinates	width (µm)
L1	(12.39, 21.41)	0,61
L2	(10.69, 19.21)	0,24
L3	(12.19, 19.02)	0,23
L4	(12.16, 24.18)	0,17
L5	(12.19, 26.57)	0,21
L6	(11.73, 26.72)	0,15
L7	(12.92, 27.76)	0,15
L8	(18.78, 27.28)	0,21
L9	(20.03, 23.28)	0,09
L10	(21.58, 23.74)	0,11
L11	(22.62, 23.33)	0,2
L12	(23.94, 22.43)	0,23
L13	(24.01, 21.39)	0,11
L14	(25.26, 21.78)	0,08
L15	(20.97, 21.94)	0,12
L16	(20.39, 21.01)	0,23
L17	(18.58, 28.92)	0,13
L18	(18.99, 29.11)	0,11
L19	(20.02, 29.16)	0,13
L20	(24.31, 28.45)	0,19
L21	(25.12, 27.56)	0,17
L22	(26.57, 26.79)	0,18
L23	(7.87, 28.86)	0,15
L24	(7.68, 28.01)	0,15
L25	(7.27, 27.06)	0,11
L26	(6.19, 26.22)	0,23
L27	(6.48, 25.59)	0,12
L28	(6.66, 27.91)	0,21
L29	(9.50, 28.36)	0,21
L30	(9.61, 27.01)	0,15
L31	(9.29, 26.11)	0,14
L32	(8.62, 22.53)	0,11
L33	(7.24, 22.88)	0,13
L34	(6.99, 23.24)	0,13
L35	(7.05, 24.04)	0,14
L36	(6.78, 24.95)	0,13
L37	(5.85, 25.22)	0,17
L38	(7.44, 19.46)	0,22
L39	(7.71, 18.93)	0,19
L40	(7.70, 18.12)	0,16

L41	(95.26, 37.35)	0,23	L41	(9.75, 19.46)	0,14
L42	(95.95, 36.55)	0,23	L42	(10.12, 15.89)	0,15
L43	(93.24, 35.13)	0,32	L43	(10.79, 15.09)	0,13
L44	(93.62, 34.23)	0,33	L44	(11.42, 14.43)	0,15
L45	(94.19, 32.50)	0,29	L45	(9.69, 14.36)	0,22
L46	(93.37, 31.79)	0,23	L46	(8.18, 15.53)	0,09
L47	(92.25, 31.54)	0,23	L47	(6.71, 16.32)	0,18
L48	(91.40, 33.38)	0,2	L48	(3.03, 18.28)	0,16
L49	(90.55, 33.76)	0,23	L49	(1.97, 15.79)	0,15
L50	(88.80, 33.08)	0,5	L50	(1.56, 15.41)	0,24
L51	(89.43, 35.38)	0,99	L51	(1.25, 15.64)	0,24
L52	(90.99, 35.35)	0,23	L52	(3.55, 15.79)	0,16
L53	(92.25, 37.92)	0,32	L53	(3.39, 16.16)	0,13
L54	(92.52, 40.00)	0,23	L54	(4.40, 17.14)	0,22
L55	(92.99, 41.24)	0,24	L55	(5.03, 17.31)	0,13
L56	(92.22, 42.22)	0,24	L56	(3.77, 17.29)	0,11
L57	(91.48, 42.72)	0,2	L57	(3.42, 13.97)	0,11
L58	(90.09, 42.03)	0,35	L58	(2.78, 14.13)	0,18
L59	(85.60, 42.41)	0,4	L59	(1.73, 13.55)	0,17
L60	(105.64, 37.79)	0,45	L60	(4.57, 13.15)	0,17
L61	(107.34, 37.27)	0,33	L61	(5.19, 13.40)	0,21
L62	(107.69, 36.23)	0,17	L62	(6.07, 13.76)	0,12
L63	(107.47, 35.08)	0,39	L63	(6.87, 14.05)	0,13
L64	(108.16, 32.67)	0,27	L64	(6.98, 14.33)	0,24
L65	(102.82, 33.71)	0,23	L65	(7.23, 14.66)	0,15
L66	(100.85, 34.99)	0,2	L66	(5.72, 15.55)	0,15
L67	(97.53, 32.94)	0,27	L67	(3.72, 12.39)	0,13
L68	(83.79, 31.57)	0,23	L68	(4.20, 11.93)	0,09
L69	(89.21, 28.39)	0,29	L69	(3.59, 12.12)	0,15
L70	(89.89, 29.30)	0,43	L70	(1.38, 12.48)	0,12
L71	(89.70, 30.59)	0,2	L71	(2.26, 11.68)	0,12
L72	(95.26, 29.90)	0,23	L72	(3.08, 11.15)	0,11
L73	(96.25, 27.35)	0,28	L73	(4.66, 11.13)	0,2
L74	(96.33, 25.63)	0,35	L74	(5.92, 10.88)	0,11
L75	(100.90, 27.38)	0,23	L75	(7.23, 12.18)	0,26
L76	(102.82, 28.29)	0,17	L76	(8.09, 12.46)	0,13
L77	(104.11, 27.03)	0,27	L77	(8.70, 12.28)	0,13
L78	(105.12, 24.48)	0,23	L78	(9.55, 12.08)	0,12
L79	(104.71, 24.32)	0,22	L79	(5.25, 9.99)	0,17
L80	(103.94, 24.59)	0,24	L80	(4.71, 9.74)	0,18
L81	(98.90, 24.15)	0,24	L81	(3.56, 9.95)	0,18
L82	(94.85, 23.47)	0,23	L82	(3.33, 7.18)	0,13
L83	(94.28, 23.79)	0,23	L83	(4.55, 7.44)	0,21
L84	(93.48, 24.84)	0,29	L84	(4.45, 6.99)	0,19
L85	(94.80, 25.38)	0,2	L85	(4.79, 6.35)	0,15
L86	(93.59, 27.52)	0,35	L86	(7.51, 6.74)	0,16
L87	(92.61, 29.22)	0,27	L87	(7.95, 6.38)	0,13
L88	(80.86, 29.13)	0,29	L88	(8.32, 6.13)	0,21
L89	(81.19, 32.31)	0,12	L89	(8.88, 6.79)	0,12
L90	(82.39, 34.69)	0,17	L90	(9.20, 7.33)	0,2

L91	(70.29, 34.50)	0,16	L91	(9.82, 8.66)	0,26
L92	(69.44, 34.47)	0,23	L92	(9.09, 4.36)	0,2
L93	(68.78, 33.87)	0,27	L93	(9.56, 3.88)	0,13
L94	(69.28, 35.54)	0,22	L94	(9.61, 5.52)	0,17
L95	(71.27, 35.79)	0,17	L95	(5.33, 4.75)	0,2
L96	(72.12, 35.21)	0,24	L96	(0.78, 5.74)	0,16
L97	(72.73, 34.75)	0,2	L97	(0.38, 4.73)	0,13
L98	(73.16, 29.30)	0,24	L98	(0.80, 3.42)	0,17
L99	(72.89, 27.74)	0,27	L99	(4.77, 1.10)	0,11
L100	(73.74, 26.72)	0,12	L100	(5.36, 1.15)	0,15
L101	(72.01, 27.14)	0,27	L101	(5.84, 3.23)	0,17
L102	(75.60, 25.55)	0,23	L102	(2.81, 2.05)	0,12
L103	(78.01, 27.68)	0,23	L103	(8.12, 1.10)	0,19
L104	(78.12, 29.05)	0,32	L104	(9.02, 1.56)	0,13
L105	(78.56, 31.19)	0,23	L105	(9.66, 2.46)	0,11
L106	(77.46, 33.87)	0,08	L106	(6.60, 1.19)	0,25
L107	(76.70, 35.05)	0,2	L107	(7.24, 3.60)	0,29
L108	(83.71, 35.82)	0,2	L108	(8.11, 4.43)	0,13
L109	(83.32, 35.95)	0,32	L109	(7.22, 4.73)	0,24
L110	(84.91, 34.01)	0,33	L110	(10.89, 2.21)	0,12
L111	(82.28, 24.45)	0,23	L111	(14.00, 3.70)	0,15
L112	(81.41, 23.99)	0,43	L112	(13.50, 3.59)	0,22
L113	(80.39, 24.92)	0,24	L113	(13.17, 4.67)	0,24
L114	(78.59, 24.12)	0,2	L114	(15.23, 0.73)	0,15
L115	(76.04, 24.64)	0,23	L115	(16.42, 2.25)	0,29
L116	(71.96, 25.05)	0,23	L116	(16.72, 5.16)	0,17
L117	(68.24, 25.27)	0,16	L117	(20.85, 3.89)	0,19
L118	(68.87, 26.40)	0,16	L118	(20.45, 4.47)	0,17
L119	(68.10, 27.68)	0,17	L119	(19.71, 4.12)	0,16
L120	(68.32, 29.93)	0,32	L120	(20.36, 3.18)	0,11
L121	(67.88, 31.35)	0,23	L121	(21.47, 2.43)	0,17
L122	(47.89, 33.93)	0,28	L122	(22.58, 1.82)	0,13
L123	(47.18, 33.05)	0,23	L123	(23.59, 2.22)	0,21
L124	(47.10, 32.01)	0,2	L124	(21.28, 1.60)	0,17
L125	(44.50, 31.57)	0,23	L125	(19.27, 2.16)	0,21
L126	(48.41, 28.48)	0,31	L126	(18.58, 2.61)	0,23
L127	(47.48, 27.22)	0,35	L127	(18.09, 2.08)	0,15
L128	(39.43, 29.05)	0,29	L128	(18.20, 4.34)	0,12
L129	(39.13, 32.20)	0,37	L129	(22.75, 4.32)	0,19
L130	(40.11, 32.75)	0,23	L130	(24.61, 1.86)	0,19
L131	(43.45, 35.13)	0,47	L131	(24.42, 1.00)	0,2
L132	(55.17, 32.26)	0,37	L132	(20.87, 0.56)	0,23
L133	(54.85, 32.15)	0,2	L133	(22.90, 6.37)	0,31
L134	(54.93, 31.30)	0,2	L134	(20.98, 6.45)	0,15
L135	(54.49, 31.49)	0,24	L135	(20.10, 6.16)	0,25
L136	(53.94, 31.90)	0,29	L136	(18.70, 6.17)	0,11
L137	(52.90, 32.39)	0,27	L137	(17.65, 7.86)	0,21
L138	(52.57, 31.82)	0,24	L138	(17.07, 10.50)	0,19
L139	(51.40, 31.08)	0,17	L139	(17.57, 11.15)	0,19
L140	(50.22, 31.46)	0,35	L140	(20.35, 10.74)	0,3

L141	(49.04, 31.79)	0,17	L141	(19.26, 11.11)	0,2
L142	(48.93, 33.02)	0,23	L142	(19.81, 12.49)	0,4
L143	(49.04, 35.13)	0,28	L143	(19.32, 12.19)	0,35
L144	(47.01, 36.09)	0,16	L144	(17.88, 12.38)	0,22
L145	(46.58, 35.73)	0,17	L145	(17.47, 13.77)	0,31
L146	(45.56, 36.25)	0,24	L146	(17.19, 13.01)	0,13
L147	(51.56, 20.81)	0,27	L147	(16.11, 14.55)	0,23
L148	(51.67, 21.82)	0,23	L148	(17.06, 14.99)	0,21
L149	(50.33, 21.47)	0,15	L149	(18.49, 15.20)	0,23
L150	(49.21, 23.06)	0,4	L150	(20.82, 14.82)	0,15
L151	(48.68, 21.85)	0,15	L151	(21.90, 14.53)	0,09
L152	(49.31, 21.33)	0,17	L152	(22.47, 14.79)	0,11
L153	(52.87, 19.82)	0,2	L153	(20.59, 13.11)	0,15
L154	(52.98, 23.36)	0,32	L154	(20.74, 12.79)	0,16
L155	(47.15, 24.92)	0,22	L155	(23.37, 13.10)	0,24
L156	(45.97, 24.32)	0,27	L156	(24.24, 14.25)	0,18
L157	(45.32, 23.69)	0,17	L157	(23.50, 16.06)	0,12
L158	(44.30, 22.67)	0,24	L158	(22.96, 16.05)	0,19
L159	(42.88, 22.95)	0,24	L159	(22.28, 18.02)	0,19
L160	(42.14, 21.96)	0,23	L160	(23.58, 18.20)	0,15
L161	(42.77, 20.04)	0,24	L161	(23.91, 19.06)	0,23
L162	(43.62, 18.67)	0,27	L162	(23.63, 19.56)	0,12
L163	(44.66, 20.70)	0,2	L163	(23.54, 19.74)	0,14
L164	(39.76, 21.00)	0,38	L164	(23.17, 20.14)	0,19
L165	(39.62, 20.21)	0,27	L165	(21.63, 18.37)	0,14
L166	(37.68, 21.00)	0,35	L166	(18.63, 18.17)	0,2
L167	(37.90, 22.48)	0,27	L167	(17.26, 20.30)	0,12
L168	(36.17, 22.67)	0,2	L168	(15.96, 19.92)	0,15
L169	(39.70, 24.62)	0,2	L169	(15.18, 20.70)	0,16
L170	(39.35, 24.10)	0,37	L170	(15.88, 20.94)	0,15
L171	(41.48, 23.90)	0,32	L171	(19.50, 22.30)	0,12
L172	(42.58, 24.97)	0,29	L172	(18.87, 23.34)	0,18
L173	(43.78, 25.33)	0,32	L173	(19.50, 24.11)	0,12
L174	(45.23, 26.40)	0,24	L174	(21.29, 24.53)	0,15
L175	(51.12, 26.12)	0,27	L175	(21.34, 24.94)	0,12
L176	(52.96, 25.79)	0,24	L176	(19.57, 25.74)	0,36
L177	(53.94, 26.29)	0,22	L177	(17.44, 25.12)	0,3
L178	(53.01, 24.86)	0,31	L178	(16.87, 25.44)	0,13
L179	(52.38, 24.18)	0,23	L179	(16.50, 25.51)	0,08
L180	(56.43, 24.04)	0,27	L180	(14.42, 23.13)	0,39
L181	(57.83, 23.47)	0,2	L181	(3.35, 25.89)	0,31
L182	(56.84, 22.56)	0,22	L182	(3.07, 25.41)	0,21
L183	(54.87, 21.60)	0,27	L183	(4.31, 25.34)	0,09
L184	(56.57, 21.25)	0,24	L184	(4.33, 26.41)	0,16
L185	(57.53, 21.93)	0,32	L185	(3.88, 26.43)	0,25
L186	(58.87, 21.30)	0,31	L186	(1.08, 26.65)	0,15
L187	(59.42, 22.04)	0,35	L187	(0.53, 26.74)	0,22
L188	(60.65, 22.86)	0,32	L188	(0.50, 23.87)	0,19
L189	(61.09, 21.11)	0,28	L189	(3.05, 23.87)	0,17
L190	(58.10, 26.23)	0,24	L190	(2.81, 24.35)	0,15

L191	(60.13, 26.72)	0,15	L191	(4.29, 21.43)	0,21
L192	(63.17, 27.71)	0,16	L192	(5.29, 21.72)	0,23
L193	(63.77, 28.34)	0,23	L193	(4.48, 19.80)	0,18
L194	(62.92, 29.82)	0,44	L194	(6.92, 21.74)	0,2
L195	(64.07, 31.63)	0,2	L195	(1.29, 21.13)	0,15
L196	(61.66, 31.22)	0,11	L196	(1.45, 20.97)	0,13
L197	(57.34, 32.53)	0,24	L197	(0.81, 20.42)	0,19
L198	(52.76, 30.78)	0,2	L198	(0.31, 20.31)	0,19
L199	(49.83, 29.65)	0,35	L199	(1.51, 19.85)	0,23
L200	(49.40, 29.16)	0,23	L200	(29.82, 3.44)	0,11
**Average**		**0,25**	**Average**		**0,18**

	**TN8**	
measure	coordinates	width (µm)
L2	(3,74, 2,97)	0,46
L4	(3,40, 2,66)	0,12
L6	(4,30, 2,67)	0,12
L8	(4,14, 2,23)	0,15
L10	(4,25, 2,46)	0,07
L12	(3,70, 1,39)	0,22
L14	(1,97, 2,35)	0,32
L16	(1,06, 1,86)	0,25
L18	(1,56, 2,00)	0,24
L20	(1,32, 1,07)	0,27
L22	(0,66, 1,45)	0,22
L24	(1,82, 1,00)	0,17
L26	(1,57, 1,15)	0,07
L28	(2,72, 1,18)	0,28
L30	(4,68, 3,58)	0,12
L2	(2,48, 1,72)	0,12
L4	(1,55, 2,07)	0,14
L6	(1,71, 2,23)	0,25
L2	(1,64, 1,76)	0,11
L4	(1,82, 1,99)	0,11
L6	(2,56, 1,59)	0,13
L8	(1,56, 0,69)	0,09
L10	(3,61, 0,58)	0,32
L12	(3,03, 1,48)	0,12
L14	(2,42, 2,54)	0,1
L16	(3,00, 2,32)	0,14
L18	(1,91, 2,61)	0,13
L20	(2,45, 2,09)	0,18
L22	(3,34, 2,14)	0,15
L24	(3,24, 1,94)	0,12
L26	(4,76, 1,23)	0,19
L28	(4,52, 1,84)	0,21
L30	(3,87, 2,66)	0,12
L32	(4,28, 3,02)	0,13
L34	(5,00, 2,70)	0,24
L2	(2,35, 2,92)	0,15
L4	(1,34, 1,17)	0,16
L6	(1,02, 1,66)	0,09
L8	(1,17, 1,95)	0,11
L10	(1,96, 1,52)	0,12

L12	(3,04, 1,67)	0,26
L14	(2,51, 2,60)	0,12
L16	(3,94, 2,12)	0,12
L18	(3,18, 0,88)	0,09
L20	(3,00, 0,73)	0,11
L22	(2,63, 0,69)	0,13
L24	(2,17, 1,16)	0,11
L26	(0,91, 0,77)	0,24
L2	(1,71, 1,50)	0,14
L2	(3,69, 2,72)	0,11
L4	(3,08, 2,19)	0,14
L6	(2,96, 1,97)	0,13
L8	(2,41, 2,39)	0,14
L10	(1,45, 1,90)	0,11
L12	(1,57, 2,09)	0,11
L14	(2,77, 2,87)	0,1
L16	(2,03, 2,78)	0,11
L18	(1,58, 0,49)	0,1
L20	(2,14, 2,16)	0,18
L22	(1,67, 2,70)	0,16
L24	(3,37, 0,54)	0,22
L26	(4,18, 2,21)	0,17
L2	(1,52, 1,34)	0,17
L4	(1,91, 1,27)	0,24
L6	(2,09, 0,58)	0,18
L10	(12,82, 5,32)	0,16
L26	(6,10, 11,38)	0,14
L34	(1,86, 7,34)	0,15
L42	(7,29, 1,59)	0,29
L46	(10,56, 2,04)	0,24
L50	(13,00, 1,88)	0,11
L54	(11,66, 3,18)	0,18
L58	(10,99, 2,99)	0,22
L60	(15,26, 4,09)	0,19
L62	(14,30, 2,27)	0,16
L64	(15,66, 2,66)	0,09
L66	(17,19, 4,80)	0,13
L68	(16,72, 3,78)	0,13
L70	(17,65, 4,02)	0,14
L72	(18,52, 1,79)	0,1
L74	(18,44, 5,70)	0,18
L76	(19,15, 6,95)	0,17
L78	(18,95, 7,17)	0,15
L80	(12,88, 11,60)	0,17
L82	(12,29, 11,46)	0,31
L84	(10,61, 11,56)	0,29
L86	(9,44, 12,45)	0,28
L88	(8,58, 11,82)	0,24
L90	(9,99, 14,89)	0,11
L92	(14,39, 13,33)	0,12

L94	(11,25, 3,77)	0,15
L96	(5,30, 7,49)	0,38
L98	(8,35, 8,86)	0,2
L100	(8,78, 8,64)	0,1
L102	(6,65, 11,07)	0,11
L2	(5,18, 2,39)	0,12
L4	(3,18, 1,46)	0,15
L6	(1,97, 0,55)	0,22
L8	(4,87, 1,57)	0,14
L10	(6,08, 1,38)	0,11
L12	(6,66, 1,34)	0,19
L14	(6,87, 2,42)	0,21
L16	(1,48, 5,47)	0,17
L18	(1,69, 6,14)	0,09
L20	(1,10, 6,47)	0,16
L22	(1,25, 7,23)	0,08
L24	(6,11, 6,33)	0,17
L26	(6,77, 5,18)	0,14
L28	(8,19, 5,89)	0,13
L30	(8,34, 5,83)	0,11
L32	(8,81, 6,05)	0,18
L34	(8,21, 7,38)	0,21
L36	(8,28, 7,04)	0,17
L38	(12,06, 6,43)	0,17
L40	(11,74, 7,27)	0,23
L42	(11,56, 7,11)	0,17
L44	(9,11, 7,81)	0,22
L46	(7,79, 4,75)	0,29
L48	(6,84, 4,01)	0,18
L50	(1,77, 4,72)	0,15
L52	(1,01, 3,85)	0,13
L54	(2,79, 5,69)	0,16
L56	(3,51, 5,17)	0,13
L58	(2,92, 6,38)	0,13
L60	(0,15, 1,96)	0,07
L62	(9,30, 2,54)	0,13
L64	(8,70, 3,03)	0,11
L66	(5,77, 0,38)	0,16
L68	(2,95, 0,88)	0,31
L70	(0,63, 2,50)	0,16
L72	(1,44, 2,53)	0,15
L74	(3,40, 3,31)	0,1
L76	(11,33, 1,28)	0,17
L78	(11,10, 6,17)	0,19
L80	(10,42, 6,47)	0,1
L82	(9,27, 7,12)	0,22
L84	(6,99, 5,74)	0,16
L86	(1,78, 7,79)	0,14
L2	(5,02, 2,96)	0,22
L4	(2,49, 1,42)	0,1

L6	(5,27, 1,01)	0,09
L8	(2,02, 6,72)	0,12
L10	(1,38, 6,61)	0,13
L12	(8,82, 5,44)	0,19
L14	(4,60, 7,34)	0,14
L16	(4,73, 7,58)	0,12
L18	(3,89, 1,85)	0,07
L2	(7,49, 5,70)	0,09
L4	(5,97, 5,33)	0,11
L6	(5,04, 5,76)	0,18
L8	(5,70, 4,05)	0,15
L10	(3,44, 1,85)	0,1
L12	(4,63, 3,99)	0,12
L14	(5,31, 3,94)	0,18
L16	(4,22, 2,99)	0,24
L18	(1,44, 2,31)	0,1
L20	(7,11, 2,17)	0,17
L22	(4,79, 0,82)	0,2
L24	(1,89, 4,68)	0,33
L26	(1,03, 4,86)	0,19
L28	(2,30, 0,52)	0,13
L30	(4,89, 2,49)	0,21
L32	(6,40, 2,65)	0,07
L2	(5,03, 2,31)	0,18
L4	(5,69, 3,62)	0,3
L6	(2,24, 2,80)	0,13
L8	(1,71, 2,47)	0,32
L10	(1,61, 4,19)	0,12
L12	(0,98, 4,24)	0,14
L14	(1,17, 2,37)	0,16
L16	(1,82, 1,20)	0,19
L18	(3,51, 0,86)	0,16
L24	(5,83, 11,15)	0,22
L56	(36.61, 29.16)	0,46
L57	(46.42, 33.28)	0,12
L58	(7.32, 37.39)	0,12
L59	(6.79, 38.27)	0,15
L60	(5.19, 33.28)	0,07
L61	(8.73, 31.77)	0,22
L62	(13.85, 30.49)	0,32
L63	(12.91, 30.69)	0,25
L64	(2.70, 28.81)	0,24
L65	(3.17, 26.30)	0,27
L66	(3.05, 23.74)	0,22
L67	(3.97, 20.97)	0,17
L68	(4.89, 22.29)	0,07
L69	(11.55, 21.28)	0,28
L70	(13.75, 17.98)	0,12
L71	(7.18, 7.81)	0,12
L72	(13.95, 5.94)	0,14

L73	(19.93, 1.19)	0,25
L74	(17.65, 1.77)	0,11
L75	(23.00, 2.72)	0,11
L76	(1.42, 8.04)	0,13
L77	(20.95, 7.22)	0,09
L78	(19.58, 14.52)	0,32
L79	(5.50, 14.56)	0,12
L80	(13.30, 28.08)	0,1
L81	(23.80, 23.29)	0,14
L83	(26.10, 31.49)	0,18
**Average**		**0,16**
